# Molecule auto-correction to facilitate molecular design

**DOI:** 10.1007/s10822-024-00549-1

**Published:** 2024-02-16

**Authors:** Alan Kerstjens, Hans De Winter

**Affiliations:** Laboratory of Medicinal Chemistry, Department of Pharmaceutical Sciences, University of Antwerp, Universiteitslaan 1, 2610 Wilrijk, Belgium

**Keywords:** Molecular design, Auto-correct, Tree search, Policy, Perturbation

## Abstract

Ensuring that computationally designed molecules are chemically reasonable is at best cumbersome. We present a molecule correction algorithm that morphs invalid molecular graphs into structurally related valid analogs. The algorithm is implemented as a tree search, guided by a set of policies to minimize its cost. We showcase how the algorithm can be applied to molecular design, either as a post-processing step or as an integral part of molecule generators.

**Graphical abstract:**

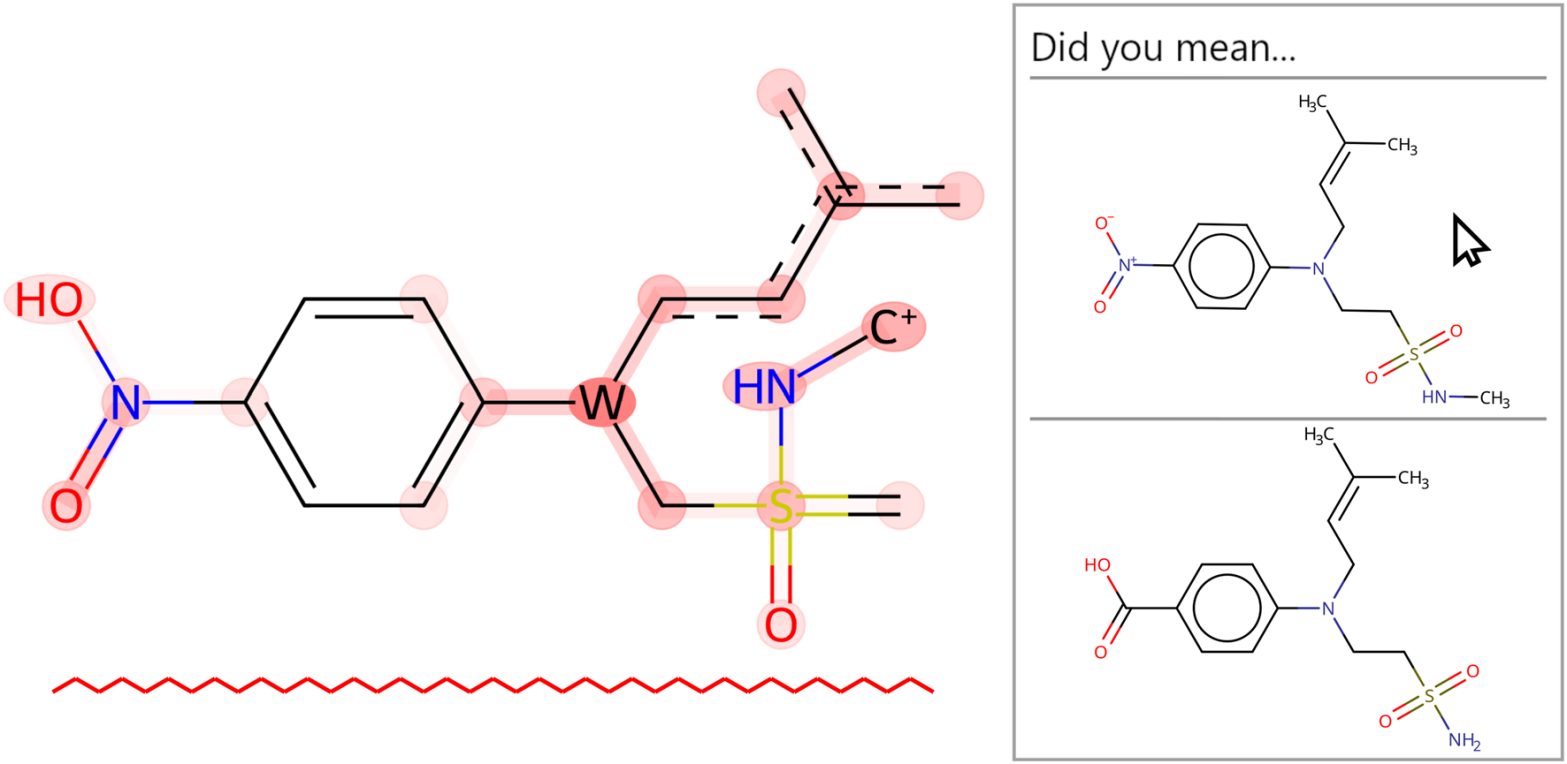

**Supplementary Information:**

The online version contains supplementary material available at 10.1007/s10822-024-00549-1.

## Introduction

**Fig. 1:**
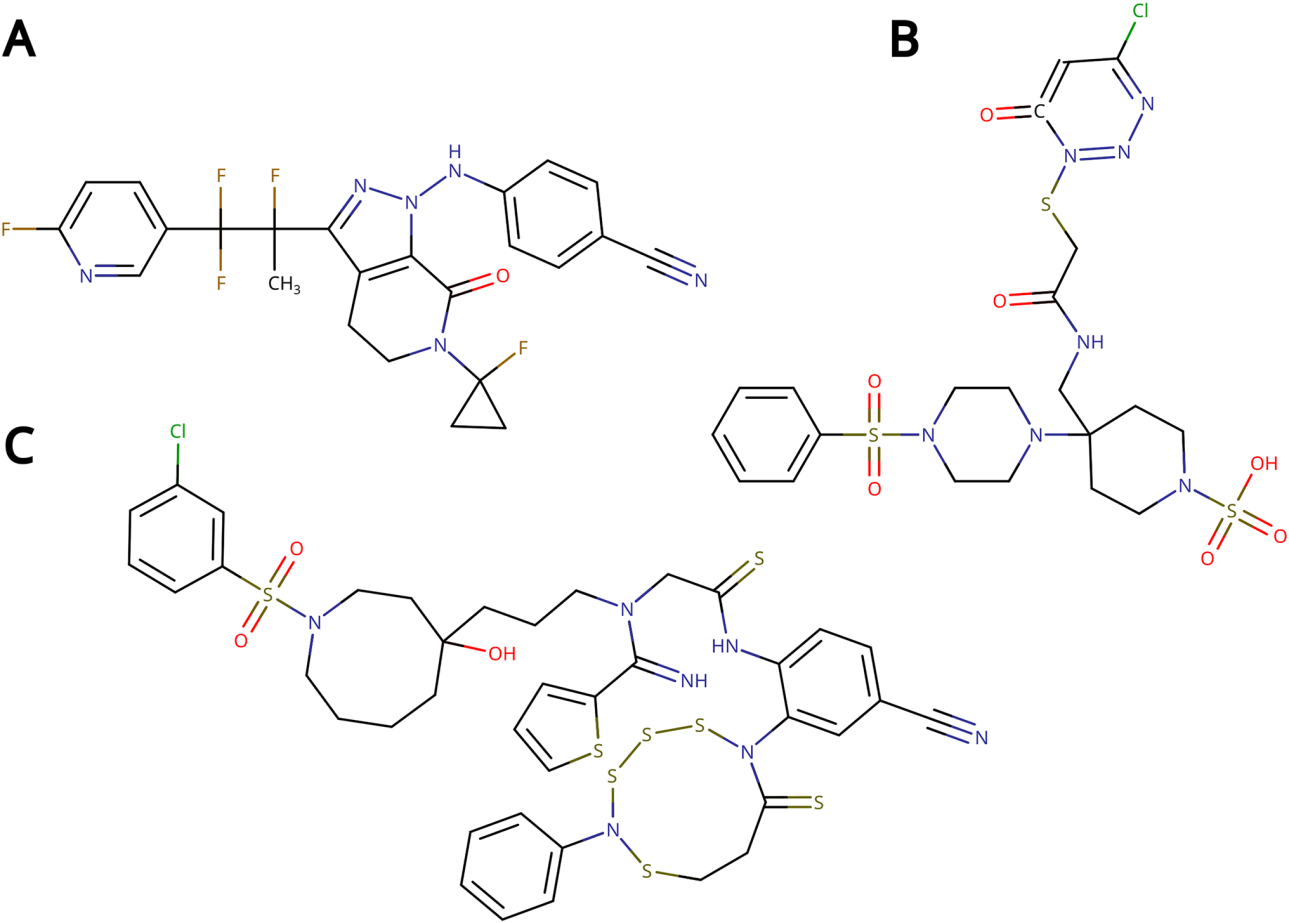
*Examples of objectionable molecules generated by diverse molecule generators during a JAK2 inhibitor design exercise, as reported by *[4]*. A was generated by a graph-based genetic algorithm *[6]*, ****B was generated by a particle swarm optimizer in an auto-encoder latent space ***[7]* and C was generated by a SMILES-based recurrent neural network *[8]

Computational drug discovery is a challenging task. Not only must a computational chemist strive to design molecules with potent predicted biological activity, but they must also ensure that the designed molecules are “reasonable”, to the extent that they are chemically valid, synthesizable, and overall drug-like. Experienced chemists will know a problematic molecule when they see one [1, 2]. Unfortunately, virtual molecule generators, lacking chemical intuition, tend to propose molecules that are chemically unstable and reactive, difficult to synthesize, conformationally strained or exhibit impossible electronic configurations [3–5] (Fig. 1).

Medicinal chemistry efforts are focused on designing chemically attractive molecules, which wind up recorded in chemical databases [9–11]. The “similar structure, similar property” principle, which is the cornerstone of molecular design, claims that similar molecules exhibit similar properties [12, 13]. It follows that if a query molecule resembles known appealing molecules it is likely to be appealing itself, with the contrary being the case when it is dissimilar.

The similarity principle can be exploited to reduce the likelihood of designing uncomely molecules. Indeed, most molecular design algorithms rely on it in some shape or form. A historically popular approach has been to construct chemicals as combinations of smaller molecular fragments, either systematically extracted from reference molecules [14–18] or sourced from commercial reagent libraries [19, 20]. Fragment combination is governed by rules that range in chemical sophistication from knowledge-based bonding [15–17] to simulated chemical reactions [18–20]. More recent research efforts have focused on generative models, that is, machine learning models trained to learn chemical distributions and sample molecular representations from them. The most popular molecular representation for generative models are text-based line notations [21–23]. Models can learn the syntax and semantics of said line notations, allowing them to translate back and forth between discrete textual and continuous numerical molecular representations [7, 24, 25] or to generate strings character-by-character conditioning the character probability distribution on previously sampled characters [8, 26, 27]. Alternative approaches consider chemical appeal as explicit parameters in a multi-objective optimization setting [28, 29], but even then the similarity principle frequently makes an appearance as the basis of chemical desirability objective functions [30–32].

The aforementioned strategies succeed at reducing the number of deficient generated molecules to varying degrees, but they are not infallible [3, 5]. Many resources are being channeled into further developing techniques to generate chemically desirable molecules. An unfortunate casualty of this focused effort is that research into optimization and search strategies has been stalled.

Herein we describe an algorithm that “corrects” molecules. Our intention is twofold. Firstly, we envision the tool being used to address molecule quality issues that were not caught or covered by a third-party molecule generator. Secondly, and perhaps most importantly, we hope that it will enable researchers to divest some of their attention from avoiding non-sense molecule generation to other aspects of molecular design.

The algorithm describes a query molecule with local structural features and compares said features to those found in reference desirable molecules. If the query molecule possesses features that are absent or rare in the reference molecules, the features are deemed “foreign” or incorrect. Otherwise, they are deemed “familiar” or correct. Through a tree search algorithm, we locally modify foreign features until they are familiar enough. Certain heuristics are used to prioritize modifications that are most likely to yield familiar features. One can draw an analogy between our algorithm and a primitive spell checker, where chemical features are the equivalent of words. Each word is checked against a dictionary of known words. If a word is not present, it is deemed incorrect and a heuristic suggests similar correct words.

## Methods

### Molecular characterization

**Fig. 2:**
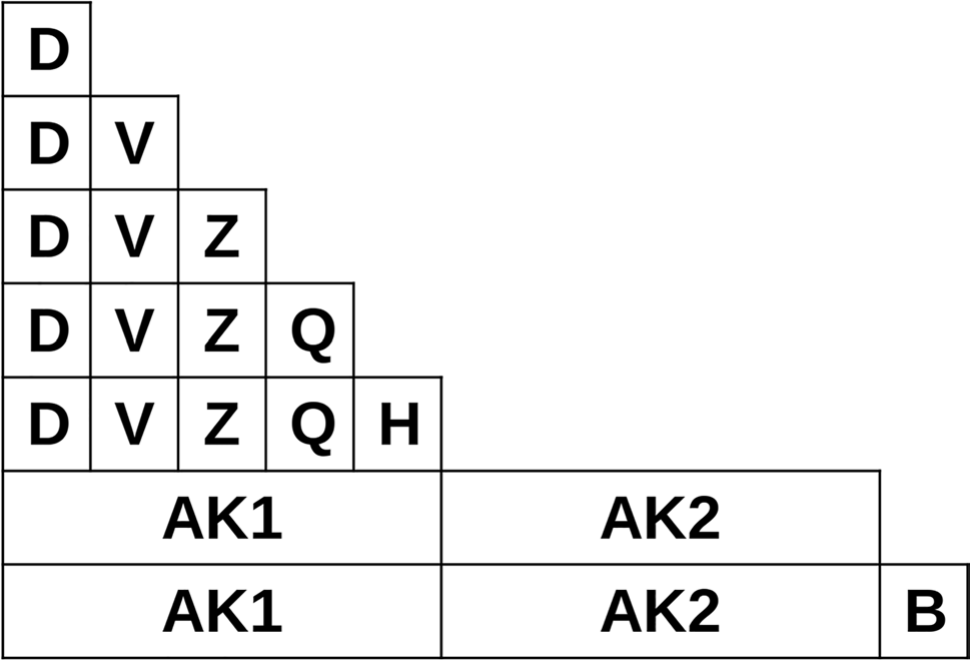
Partial atom and bond key pyramid. Higher order keys encompass lower order keys. The (D, V, Z, Q, H) key constitutes the atom key AK, and (AK1, AK2, B) constitutes the bond key

To identify if a molecule is foreign, and if so, what parts are foreign, we defined some simple localized molecular descriptors. Atoms were characterized with atom keys. Atom keys are integer tuples comprising an atom’s degree (D) (i.e. its number of adjacent atoms), valence (V), atomic number (Z), formal charge (Q) and number of hydrogens (H). These properties were chosen because they are largely independent from the atom’s surrounding chemical environment. To avoid cyclic dependencies between properties, in this work valence is defined as the sum of an atom’s bonds’ orders, without considering the atom’s formal charge. The order of the atom key’s properties is relevant. We ordered the properties by perceived decreasing significance or importance. For example, we assume that a change in degree, and therefore topology, is more disruptive to a molecule’s structure and properties than a change in atomic number. Bonds were characterized as a tuple of the bonded atoms’ keys (AK) and an integer representing the bond’s type (B), which can be thought of as the bond’s order (Fig. 2).

We also defined partial keys of the atom and bond keys. Partial atom keys were constructed by taking the first *j* most significant properties of the atom key, with *j∈  [1, k-1]*, where *k* is the number of properties in an atom key. Consequently, partial key* j* contains all partial keys with a lower* j*. The same procedure was applied to bond keys but with the range *j ∈ [2, k-1]*. This yields a total of four partial atom keys and one partial bond key (Fig. 2). Partial keys can be sorted lexicographically, enabling fast key-value store searches.

Lastly, circular atomic environments were defined for all atoms in the molecules. A circular atomic environment comprises a central atom and all surrounding atoms within a given topological distance termed the environment’s radius *r*. The resulting atomic environment was hashed to an integer using the Morgan algorithm [33, 34], and said hash was taken as the environment key or unique identifier of the environment. As such, these identifiers are conceptually equivalent to ECFP fingerprint features [34]. The Morgan algorithm requires initial atom identifiers or “invariants”. There is some flexibility in the selection of atomic invariants. By default we use the atom keys’ hashes as invariants, mimicking the Daylight atomic invariants [35]. However one could incorporate other information such as ring membership [34, 36].

### Reference dictionary

In this work a subset of ChEMBL31 [10] was chosen as the reference library of drug-like molecules. Only small organic molecules were retained. Large biomolecules, natural products and polymers were excluded. For the remaining molecules the unsalted and non-ionized “parent form” was chosen. Molecules in the reference library were characterized using the aforementioned descriptor keys, and the frequency of each key recorded in a “chemical dictionary”. We generated two dictionaries using environment radii of 1 and 2 respectively. If a key’s frequency surpasses a user-specified threshold (by default 0) it is deemed familiar, and otherwise it is deemed foreign. Owing to the way in which keys are defined, simpler keys are contained by more complex keys. For example, environment keys contain bond keys and bond keys contain atom keys (Fig. 2). This defines unidirectional dependency relationships between them, meaning that if a key is foreign all dependent keys containing it must also be foreign. The reverse is not necessarily true.

### Tree search algorithm

The molecule correction algorithm was implemented as a tree search. An incorrect input molecule serves as the root of the tree. With each iteration a molecule or vertex within the tree is selected and partially expanded. Expansion in this context means enumeration of topologically similar neighboring molecules, and establishment of a parental relationship between the selected predecessor and its neighboring successors. Expansions were performed using the graph-based molecule perturbation library Molpert [36]. Perturbations performed by the library include atom- and bond invariant changes and atom/bond insertions/deletions. To expedite the correction process molecules are sanitized (as described in [36]) after each perturbation by default, but this behavior can be disabled. Molpert enables the systematic enumeration of a molecule’s neighbors. Neighbors are enumerated lazily. The enumeration order is optimized to maximize the likelihood of finding a correct molecule with the smallest number of expansions.

As with any tree search algorithm, the search is guided by a search strategy or *policy* that dictates how the tree is expanded with each iteration. For our tree search we distinguish two different types of policies. One policy, which we call the *selection policy*, selects which vertex to expand next. The second policy, termed the *expansion policy*, determines how the selected vertex is expanded.

#### Selection policy

To guide the search towards familiar molecules we define the concept of *familiarity*. Every time a vertex is added to the tree it is featurized into atom, bond, and environment keys. Said keys are classified into foreign and familiar by looking them up in the chemical dictionary. Familiarity is calculated as a function of the total number of keys n (Eq. 1) and the number of familiar keys n^f^ (Eq. 2).

1 $$n={n}_{a}+{n}_{b}+{n}_{e}$$

2 $${n}^{f}={n}_{a}^{f}+{n}_{b}^{f}+{n}_{e}^{f}$$

In Eq. 1 n_a_, n_b_ and n_e_ denote the total number of atom, bond, and environment keys of a given molecule respectively, whereas in Eq. 2 n^f^_a_, n^f^_b_ and n^f^_e_ denote their familiar counterparts.

We employ two alternative definitions of familiarity: *f*_*1*_ (Eq. 3) and *f*_*2*_ (Eq. 4). Both range between 0 and 1, with 1 indicating a familiar or correct molecule, and can mostly be used interchangeably. *f*_*1*_ can be interpreted as a similarity coefficient between a query molecule and some unknown correct molecule. Conversely, *1—f*_*1*_ can be interpreted as the distance to a correct molecule. *f*_*1*_ is therefore well suited for estimating how close to a solution a given molecule is. *f*_*2*_ provides weaker theoretical guarantees as a similarity coefficient, for its lower boundary is dependent on the molecule’s size. *f*_*1*_’s drawback is that it can be maximized trivially by incrementing the numerator and denominator by the same amount, as occurs when adding new familiar environments (e.g. alkane carbons). *f*_*2*_ cannot be exploited in the same way, and is better suited as an optimization target.

3 $${f}_{1}=\frac{{n}^{f}}{n}$$

4 $${f}_{2}=\frac{1}{n-{n}^{f}+1}$$

**Fig. 3:**
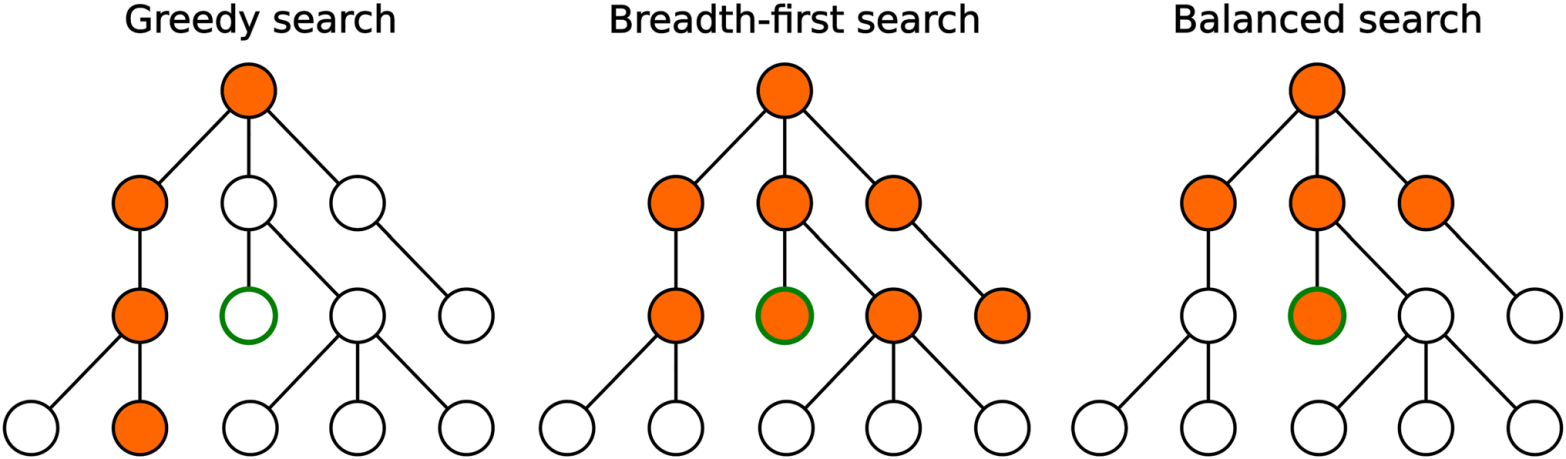
Different types of selection policies. Orange vertices represent visited vertices. The goal is to find the optimal green vertex while minimizing the number of visited vertices. Greedy search visits very few vertices but may miss the goal vertex. Breadth-first search is guaranteed to find the goal vertex but visits many other vertices in the process. An ideal selection policy balances exploration and exploitation

Different selection policies were explored. In all cases the selection is limited to foreign molecules that have not been fully expanded yet. As baselines we evaluated Breadth-First Search (BFS), where the shallowest vertices are expanded first, and greedy familiarity selection, where the vertices with the highest *f*_*2*_ familiarity are expanded first. These correspond to exploration-only and exploitation-only approaches respectively (Fig. 3). Note that a deep BFS is computationally intractable since the branching factor of chemical space is very large (Figure S1, Additional file 1).

There are many correct molecules and many paths leading to them from the input molecule. We would prefer finding the correct molecule *w* that is most closely related to the input or root molecule *u*, as according to the similar property principle it is the most likely to preserve the properties of the input molecule. The distance between the input molecule *u* and another molecule *v* of the tree is measured as the ECFP4 Tanimoto distance *d(u,v)* between both. We chose this fingerprint and distance metric combination because they have been shown to be good predictors of activity preservation [37–39].

Some policies to favor shallow tree searches and better balance exploration and exploitation were devised (Fig. 3). The naivest one is to greedily select vertices with the highest *f*_*1*_*/d(u,v)* ratio. More sophisticated policies are described below.

##### Upper confidence bounds applied to trees

One can estimate how close a vertex is to a yet to be discovered correct molecule using the familiarity metric. However, it is not always true that the vertex with the highest familiarity is involved in the shortest path to the closest correct molecule. The values (i.e. familiarities) of a parent vertex’s children follow an a priori unknown distribution. We can get better estimates of the expected child value by sampling or generating more children. As more samples become available the estimate trends towards the true value. Given limited computational resources one must choose between exploring vertices with uncertain distributions or exploiting vertices with the most promising distributions. This is known as a bandit problem, and the Upper Confidence Bound (UCB) strategy can be applied to tackle it [40]. UCB applied to Tree searches (UCT) dictates that at each iteration one should expand the vertex with the highest upper confidence interval bound [41]. In other words, one should expand the vertex for which the potential upside is maximized. Mathematically, this means expanding the vertex *v* maximizing Eq. 5.

5 $$UCB1=\overline{{f }_{1v}}+c\sqrt{\frac{ln\left({N}_{v}\right)}{{n}_{v}}}$$

In Eq. 5$\overline{{f }_{1v}}$ is the average *f*_*1*_ familiarity of *v*’s children, *n*_*v*_ is the number of times *v* was expanded, *N*_*v*_ is the number of times *v*’s parent was expanded. The first term of Eq. 5 is exploitative and the second term is explorative. *c* is a coefficient balancing between exploitation and exploration. In this work we explored *c* values of ½, 1, $\sqrt{2}$ and 2.

UCT is frequently discussed in relation to Monte Carlo Tree Search (MCTS). The difference between a plain tree search and MCTS is that in the former the value of a vertex is given by a heuristic function (in our case the familiarity) whereas in the latter the value of a vertex is estimated through means of random simulations or “rollouts”. We want to clarify that our tree search is not a MCTS despite using the UCT policy, as we did not believe random simulations would produce significantly better value estimates than the familiarity heuristic and wanted to keep resource usage to a minimum.

##### A-star

The A* (pronounced A-star) search algorithm is a path finding algorithm suitable for finding close to optimal shortest paths in a graph within reasonable amounts of time [42]. It selects for exploration/expansion the vertex *v* for which Eq. 6 is minimized.

6 $$g\left(v\right)=m\left(v\right)+h\left(v\right)$$

In Eq. 6 m*(v)* is the distance traversed to reach *v*. In our case *m(v)* is the topological distance between vertex *v* and the root vertex *u*, that is, *m(v)* = *d(u,v)*. *h(v)* is a heuristic estimate of the distance between *v* and an end point *w*, in our case a correct molecule. In other words, *h(v)* ~ *d(v,w)*. An obvious heuristic candidate is *h(v)* = *1 – f*_*1*_*(v)* (Eq. 7).

7 $$g\left(v\right)=d\left(u,v\right)+1-{f}_{1}\left(v\right)$$

*d(v,w)* is a Tanimoto distance, which is the complement of the Tanimoto similarity or Jaccard index. If *V* and *W* denote the feature set of molecules *v* and *w*, their Jaccard index *J(v,w)* is calculated according to Eq. 8.

8 $$J\left(v,w\right)=1-d(v,w)=\frac{\left|V\cap W\right|}{\left|V\cup W\right|}=\frac{\left|V\cap W\right|}{\left|V\right|+\left|W\right|-\left|V\cap W\right|}$$

*f*_*1*_*(v)* is a similarity index measuring the similarity to some unknown correct molecule *w*. While not equivalent to the Jaccard index, it is related to it. If *W* denotes the feature set of this hypothetical correct molecule, *f*_*1*_*(v)* can be rewritten as shown in Eq. 9.

9 $${f}_{1}\left(v\right)=\frac{\left|V\cap W\right|}{\left|V\right|}$$

**Fig. 4:**
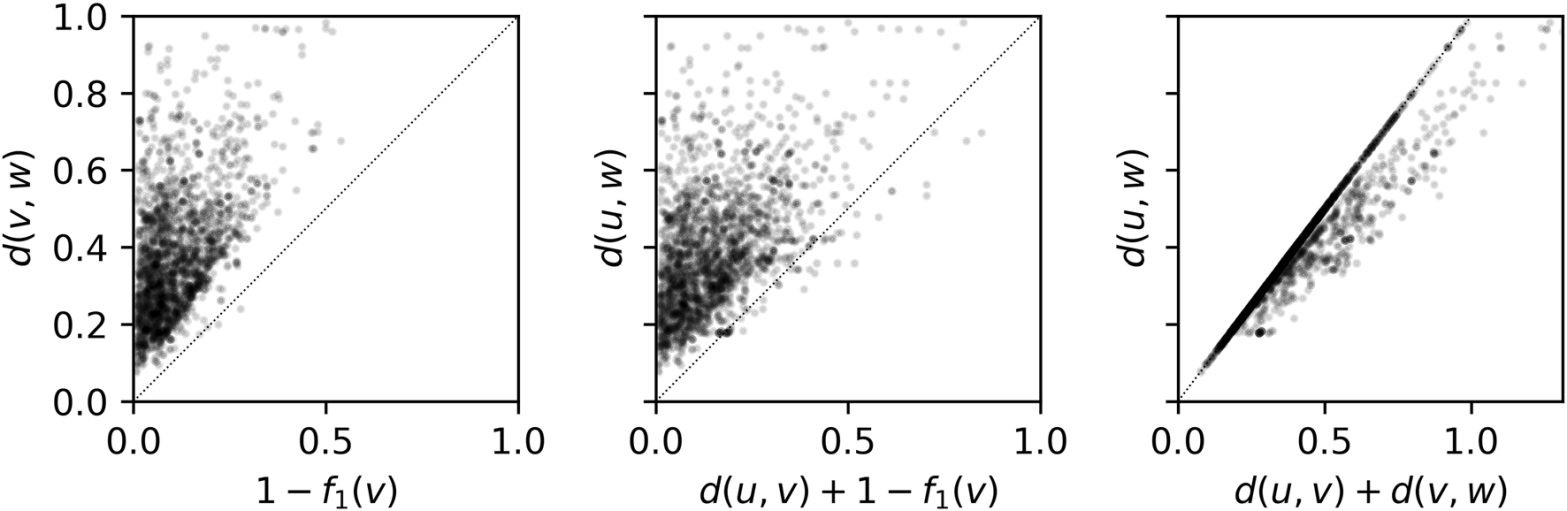
Relationships between d(u,v), d(v,w), d(u,w) and 1—f_1_(v). The two leftmost panels show that in practice 1—f_1_(v) is an almost admissible and consistent heuristic respectively. The rightmost panel is visual proof of Jaccard distances obeying the triangle inequality. Note that the correlation between d(u,v) + d(v,w) and d(u,w) is very high, which is typical of hyper dimensional spaces such as chemical space

If *f*_*1*_*(v)* were calculated using as keys solely ECFP features Eq. 8 and Eq. 9 would differ only in their denominator. It is clear that $\left|V\right|+\left|W\right|-\left|V\cap W\right|\ge \left|V\right|$. Therefore, *1 – d(v,w)* ≤ *f*_*1*_*(v)*, or equivalently *1 – f*_*1*_*(v)* ≤ *d(v,w)*, which would make *1 – f*_*1*_*(v)* an admissible heuristic. Moreover, since Jaccard distances are known to satisfy the triangle inequality [43], that is, *d(u,w)* ≤ *d(u,v)* + *d(v,w),* the heuristic would also be consistent. Using a consistent heuristic guarantees that the algorithm will find the optimal solution given enough time. We included additional terms in *f*_*1*_*(v)* besides the environment keys as we believe this additional granularity can provide finer guidance to the tree search. Consequently *1 – f*_*1*_*(v)* as described in Eq. 3 is theoretically not an admissible heuristic. Nonetheless in practice it very rarely overestimates the *d(v,w)* distance (Fig. 4).

##### Multiple linear regression distance prediction

**Fig. 5:**
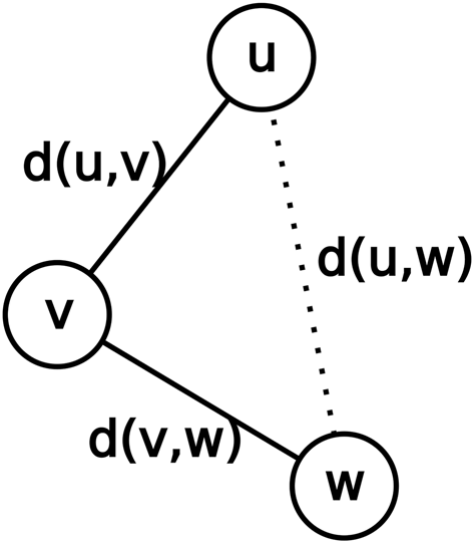
Difference between path distance (d(u,v) + d(v,w)) and straight distance (d(u,w))

A* algorithm was devised for path finding and searches for the shortest path between two vertices. We are interested in finding the closest goal vertex, that is, minimizing the distance to a goal vertex “as the crow flies”. Both distances are not equivalent (Fig. 5).

**Fig. 6:**
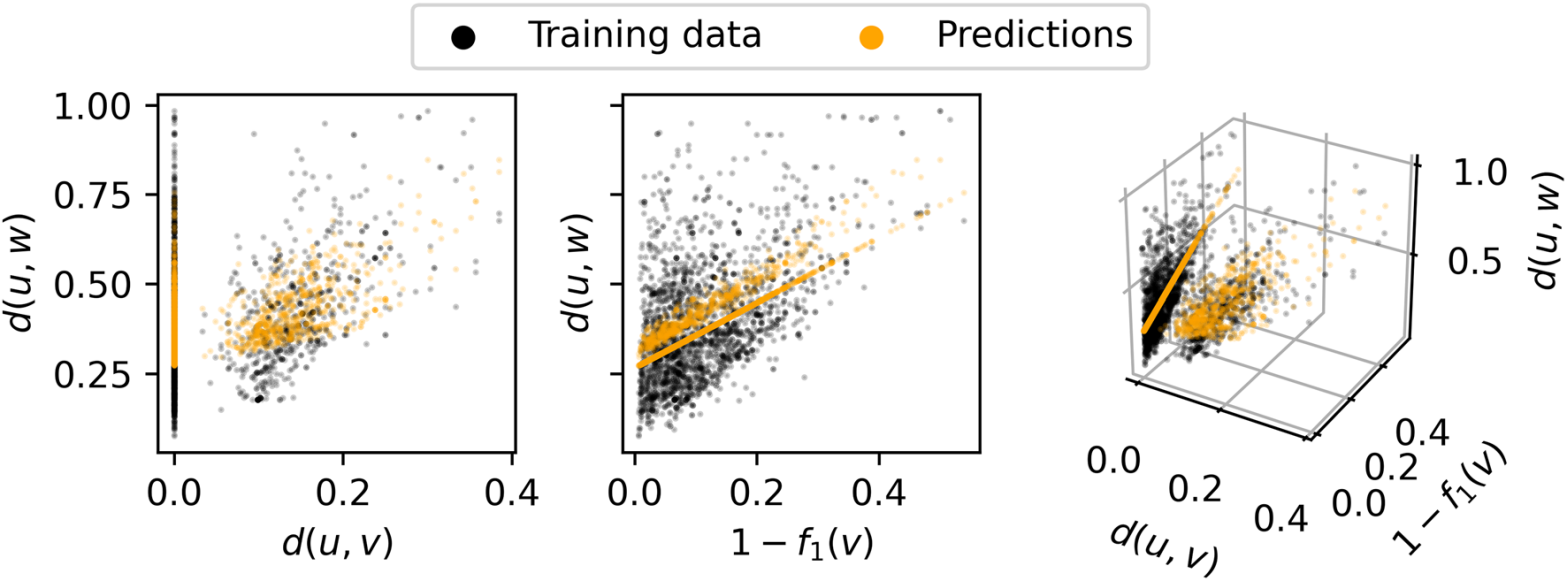
MLR model fit to training data. The two leftmost panels show the correlation between each of the model’s parameters and the training data/predictions separately, while the rightmost panel aggregates the effects of both parameters

To minimize *d(u,w)* we developed a policy that selects the vertex for which the predicted *d(u,w)* is minimal. We wanted to predict *d(u,w)* as a function of *d(u,v)* and *f*_*1*_*(v)*, which are both known for any vertex. To study the relationships between these metrics we randomly perturbed a sample of 10^3^ molecules from ChEMBL [10] by applying between 1 and 10 perturbations to each of them using Molpert [36] for a total of 10^4^ perturbed and likely incorrect molecules. We then attempted to correct these molecules with BFS as selection policy, which, given sufficient resources, guarantees to find the closest correct molecule. A dictionary containing chemical environments of radius 2 was used. Once a correct molecule had been found the search was allowed to continue until the whole tree level was visited. The maximum tree size was limited to 10^5^. Of the 10,000 structures, 1,573 molecules were successfully corrected within these resource constraints, with an average search depth of 2.4 edges. For each vertex along the shortest path between the corrected molecule and the root vertex we measured *d(u,v)*, *f*_*1*_*(v)* and *d(u,w)* for a total of 3,773 data points which we took as training data. A Multiple Linear Regression (MLR) model was fit on this data (Eq. 10), resulting in a model with a Root Mean Squared Error (RMSE) of 0.135 (Fig. 6). As a control we also built the null model $g\left(v\right)=\stackrel{-}{d(u,w)}=0.383$, with an RMSE of 0.159. Constants can be quite predictive when the response variable has a narrow range. Since our training data is comprised of shallow searches the null model appears unusually predictive. However, constants cannot extrapolate by nature, and therefore the null model won’t be predictive for deeper searches. The practical shortcomings of the null model will be showcased later.

10 $$g\left(v\right)=0.42\bullet d\left(u,v\right)-0.91\bullet {f}_{1}\left(v\right)+1.18$$

##### Explicit objective preservation

The above-described selection policies try to find correct molecules that are structurally closely related to the input molecule. The primary reason for doing so is that structurally similar molecules are believed to have similar properties [12, 13]. Yet this is not always the case [44, 45]. Two molecules may share a large common substructure and differ in a single atom. While the overall structural similarity between them may be large, if this distinctive atom is key to the molecule’s activity their properties may differ significantly.

Given an objective function *o(v)* that evaluates a vertex *v*’s property of interest we can explicitly guide the tree search into preserving this objective as opposed to relying implicitly on the similar property principle [12]. This helps tackle the cases where said principle breaks down. A simple way to do so is selecting for expansion the vertex *v* for which Eq. 11 is maximal. Note that the objectives are multiplied as opposed to being summed to prevent the search algorithm from sacrificing one objective in favor of the other.

11 $$g\left(v\right)={f}_{1}\left(v\right)\bullet o(v)$$

#### Expansion policy

A molecule is expanded by applying a perturbation to a copy of itself. Perturbations that are most likely to make the molecule familiar are applied first. Foreign molecular keys are identified and ordered according to their significance. Identifying the most significant foreign key serves as a way of identifying the most pressing problem a molecule has. The location of the problem is given by the location of the key, which is either an atom or a bond. It is this atom or bond that will be targeted by a perturbation.

When it comes to foreign atom and bond keys it is possible to identify not only the location but also the nature of the problem. Partial keys build up on each other by progressively adding properties. Since more significant keys are contained by the less significant ones the latter cannot be familiar if the former are not either. The property differentiating the most significant foreign partial key from its familiar predecessor partial key is responsible for the latter being foreign. For example, the most significant foreign partial atom key may be DVZ = (4, 6, 6), corresponding to a hexavalent carbon. Its predecessor key DV = (4, 6) is necessarily the least significant familiar key. We can then conclude that the atomic number (Z) is not compatible with the atom’s degree and valence. Since we deem the atomic number to be less significant than the degree or valence, we identify the atomic number as the culprit for the atom key being foreign, meaning perturbations modifying the atomic number will be prioritized.

The predecessor key can also be used to access the chemical dictionary and retrieve acceptable property values for the successor key. These values are sorted according to their frequency in reference molecules in descending order, meaning that the most frequent values are tried first. In the example above we can use the DV key to retrieve elements compatible with an atom of degree 4 and valence 6, which might be sulfur (Z = 16) and selenium (Z = 34). Sulfur is more frequent than selenium, so a perturbation replacing the carbon with sulfur would be prioritized.

Choosing which perturbations to apply to correct Z, Q, H or B is obvious as each of these properties has a corresponding perturbation to change its value. Correcting other properties and keys is less trivial. D is corrected by deleting bonds associated with the atom or deleting adjacent atoms. Depending on the dictionary it may also be possible to correct it by inserting more bonds or atoms, but this is disabled by default, as for organic molecules degrees higher than 6 are exceedingly rare. V is preferably corrected by changing the bond types (i.e. bond orders) of bonds associated with the atom. If this does not succeed it may also be corrected by modifying the topology of the molecule, in the same way one would correct D.

Two atom keys AK may be familiar separately, but their combination in a bond key AK1AK2 may be foreign. If the AK1AK2 partial key is foreign one or both atom keys must be changed. Perturbation types can be ordered by significance similarly to how molecular keys are ordered by significance. The lower the significance of a perturbation the less it will disrupt the molecule when applied. The perturbation significance order matches the atom property significance order (Fig. 2), being from least to most significant as follows: number of hydrogen changes, formal charge changes, atomic number changes, bond type changes, bond deletions, atom deletions, bond insertions and atom insertions. Less significant perturbations are applied first to disrupt the molecule as little as possible. While deletions do not necessarily disrupt the molecule less than insertions, they typically simplify the molecule. Simple molecules are more likely to be familiar, which is why deletions are prioritized over insertions.

**Fig. 7:**
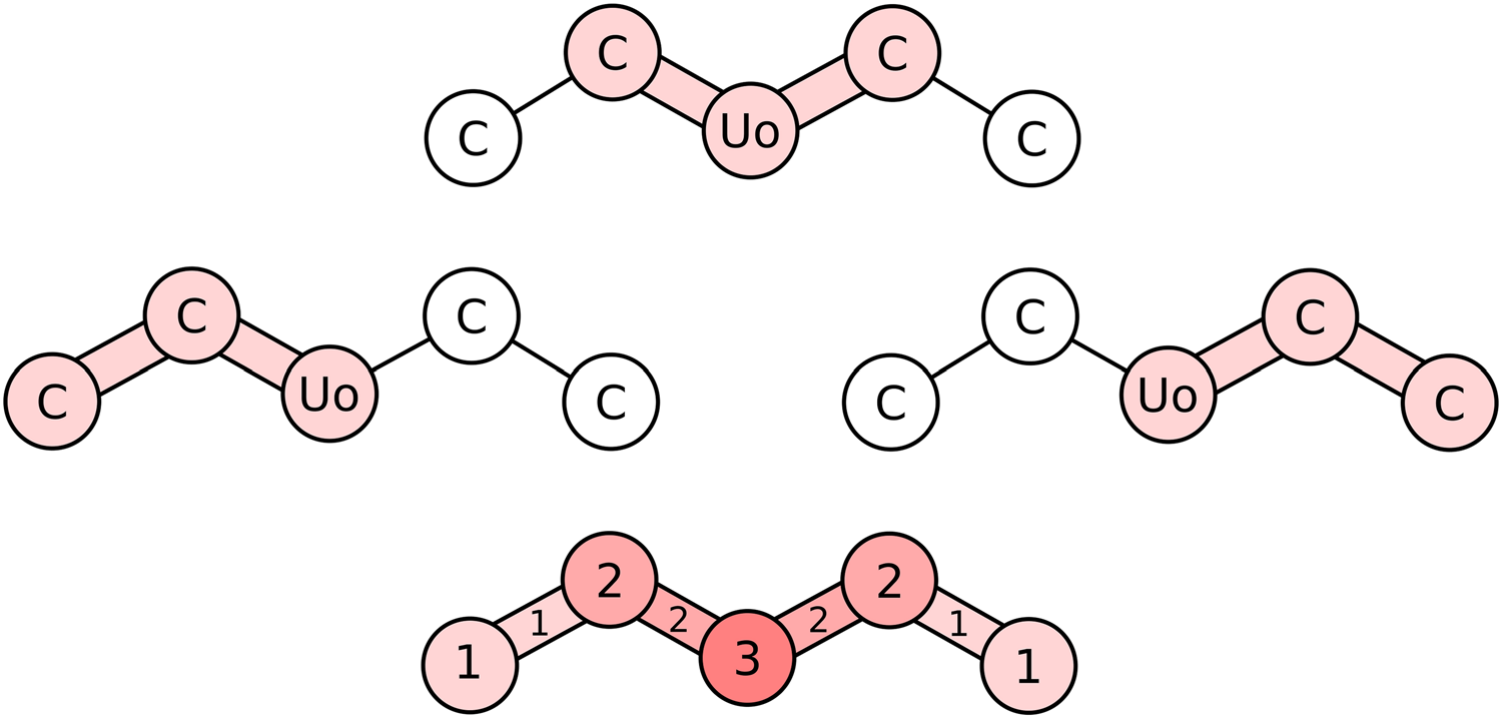
Foreign atomic environments and their overlap. The central unobtainium atom (Uo) is foreign. All atomic environments it is a part of are necessarily foreign too. Foreign circular atomic environments of radius 1 are highlighted in pink. The bottom molecule labels each atom and bond based on how many foreign environments they are involved in. The Uo atom is involved in all foreign environments, making it a likely culprit for the environments being foreign

Once all atom and bond keys have been corrected the molecule may still possess foreign atomic environments. Recall that atomic environments are characterized solely by their hash, meaning little information about what makes them foreign is available. Atomic environments overlap, in the sense that the same atom or bond may be a part of multiple environments simultaneously. Knowing the exact boundaries of atomic environments, it is possible to calculate in how many environments a given atom or bond participates (Fig. 7). We calculate the “foreign environment membership” of atoms and bonds, that is the number of foreign environments they are involved in. Atoms and bonds for which this number is highest are prioritized by perturbations, under the assumption that since they participate in many foreign environments, they are likely to be a culprit for the environments being foreign. Ties are broken with the atom- and bond keys’ frequencies, prioritizing least frequent keys. Once a target has been acquired perturbations are executed in order of increasing significance, just like for bond keys.

### Constraints

Our molecule auto-correction implementation was developed using the graph-based molecule perturbation library Molpert [36]. One of Molpert’s features is the support of user-specified arbitrary constraints perturbed molecules ought to fulfill. This functionality is inherited by the auto-correct implementation, providing the user with fine grained control over the output molecules. Among other things, this allows the user to define properties and/or parts of the molecule that should not be modified by the correction algorithm.

### Benchmark

A random sample of 10^3^ molecules from ChEMBL31 [10] was taken. Molpert [36] was used to “break” these molecules by sequentially applying 10 random perturbations to each molecule, resulting in a series of 10 perturbed and likely incorrect molecules. In total 10^4^ perturbed molecules were generated. These molecules were sorted by the number of perturbations that gave rise to them. On average, as more random perturbations are applied to a molecule, more foreign keys are generated, decreasing its familiarity. We then attempted to correct these perturbed molecules with our algorithm using the different selection policies described above. A maximum tree depth of 25 and tree size of 25,000 molecules were imposed. A chemical dictionary of circular environments of radius 2 was used for this purpose. The output molecule as well as its familiarity and similarity to the input molecule were recorded. The familiarity provides some measure of how “correct” molecules are. Nonetheless, to better contextualize the quality of the generated molecules we also measured their SAScore [30] and ran retrosynthetic analysis on them with AiZynthFinder [46] using the ZINC [11] reactants stock and United States Patent and Trademark Office-derived reaction template policy provided by the authors. SAScores were calculated using ChEMBL31 [10] as reference chemistry. Molecules were sanitized prior to calculating their properties.

**Fig. 8:**
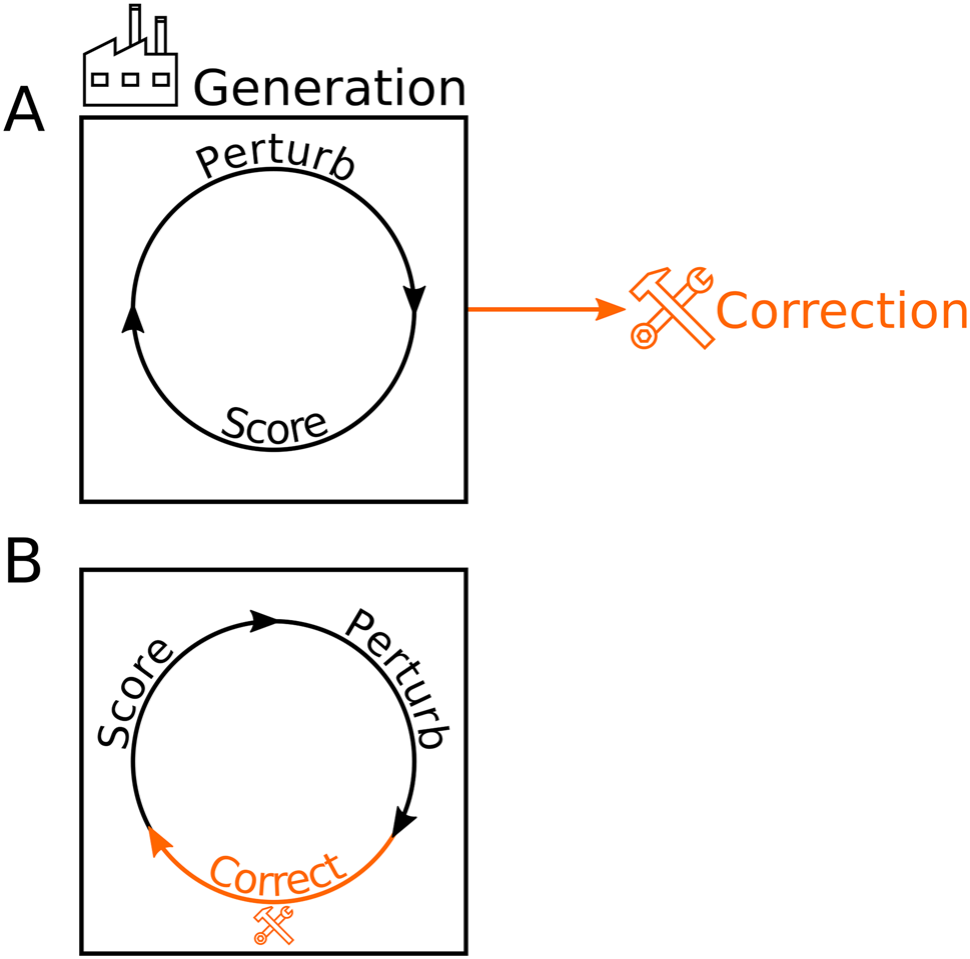
Different ways of applying molecule auto-correction in molecular design. It may be used as a final post-processing step of a molecule generator (**A**) or as an integral part of a molecule generator by injecting it into the molecule construction process (**B**)

We investigated two scenarios of how molecule correction may be applied in molecular design (Fig. 8). In both cases we took a previously published evolutionary algorithm capable of (1) designing molecules without any regard for chemical validity and (2) designing molecules fulfilling specific structural requirements [17, 36]. The algorithm was tasked with designing high-scoring molecules in the goal-directed GuacaMol benchmark suite, consisting of 20 ligand-based benchmarks [3]. As a first scenario (Fig. 8A) molecules designed without constraints by the algorithm were subjected to auto-correction as a post-processing step using different selection policies, a maximum tree depth of 25 and a maximum tree size of 25,000. For our second scenario (Fig. 8B), we injected the correction procedure as part of the mutation and recombination operators using the greedy familiarity policy, a maximum tree depth of 10 and a maximum tree size of 100. In both cases we used a chemical dictionary comprised of circular atomic environments of radius 1. 50 replicas were run for each approach, retaining the best-scoring molecule per replica and benchmark. The different approaches were compared by their designed molecules’ benchmark scores and SAScores [30]. Molecules of all 20 benchmarks and 50 replicas were aggregated, for a total of 1000 optimized molecules per approach. Benchmark scores were compared through pairwise Mann–Whitney U-tests [47] with Šidák correction [48]. SAScores were compared with Tukey’s Honestly Significant Differences test [49]. α = 0.05 was taken as family-wise error rate and significance level for all tests.

## Results and discussion

**Fig. 9:**
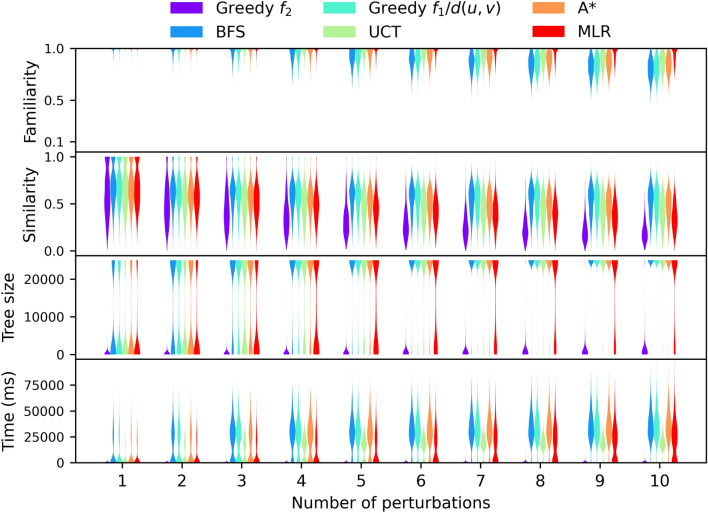
Molecule correction benchmark results. The number of perturbations applied to the input molecule is shown on the x axis. The violin plots display the density of output molecules’ properties and the cost to generate them. For the UCT policy we only display the results of using the optimal coefficient c = 0.5. Results for the remaining c values can be found in Figure S4, Additional file 1. Note that the tree size was limited to a maximum of 25,000. Timings are given for a single-threaded workload on an AMD Epyc 7452 CPU @ 2.35 GHz

Figure 9 compares the correction output using different selection policies. The amount of computational resources spent by the tree search is strongly correlated to the size of the resulting tree (Figure S2, Additional file 1). We can identify three distinct groups of policies: greedy familiarity, BFS-like policies and MLR. The greedy familiarity policy is very effective at correcting molecules, as virtually all output molecules achieve the maximum familiarity of 1 and could be considered correct. Moreover, it achieves this with a minimal amount of computational resources. Its biggest drawback, and the reason the other policies were developed, is that it favors deep searches, meaning the corrected molecules may be quite different from the input molecules.

BFS is the benchmark for how close an output molecule can possibly be to an input molecule. Indeed, unless an input molecule is familiar to begin with the output molecule must be different. Greedy distance normalized familiarity, A* and UCT approach this ceiling quite well. Unfortunately, this group of policies also spends more resources on the search, oftentimes to no avail as the output molecule is frequently not entirely familiar.

MLR stands in between the very exploitative greedy familiarity and very explorative BFS-like policies. In our opinion it achieves a good compromise between correcting molecules within reasonable amounts of time while not straying excessively far away from the input molecule. As a control we evaluated replacing the MLR model with a constant null model. Despite the null model fitting the training data well, it cannot extrapolate, leading to poor real world performance (Figure S3, Additional file 1).

**Fig. 10:**
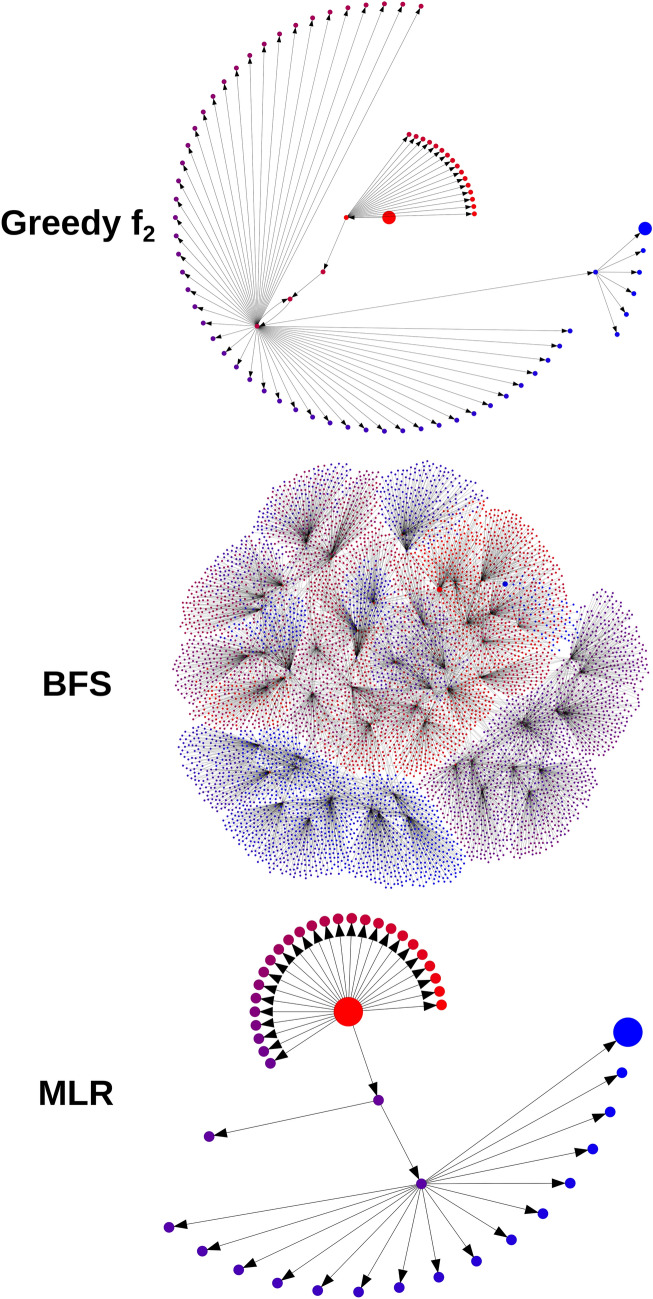
Diagrams of search trees resulting from trying to correct the same input molecule (OOC1[C]2#S1C2) using different selection policies. Nodes are color coded according to their discovery order, with red and blue being the first and last nodes to be discovered respectively. The root node is shown as a large red node, and the solution node is shown as a large blue node

To further understand the anatomy of the generated trees Fig. 10 depicts diagrams of the search trees resulting from correcting the same input molecule while using different selection policies. As can be seen the greedy *f*_*2*_ and MLR policies define narrower and deeper trees than BFS.

**Fig. 11:**
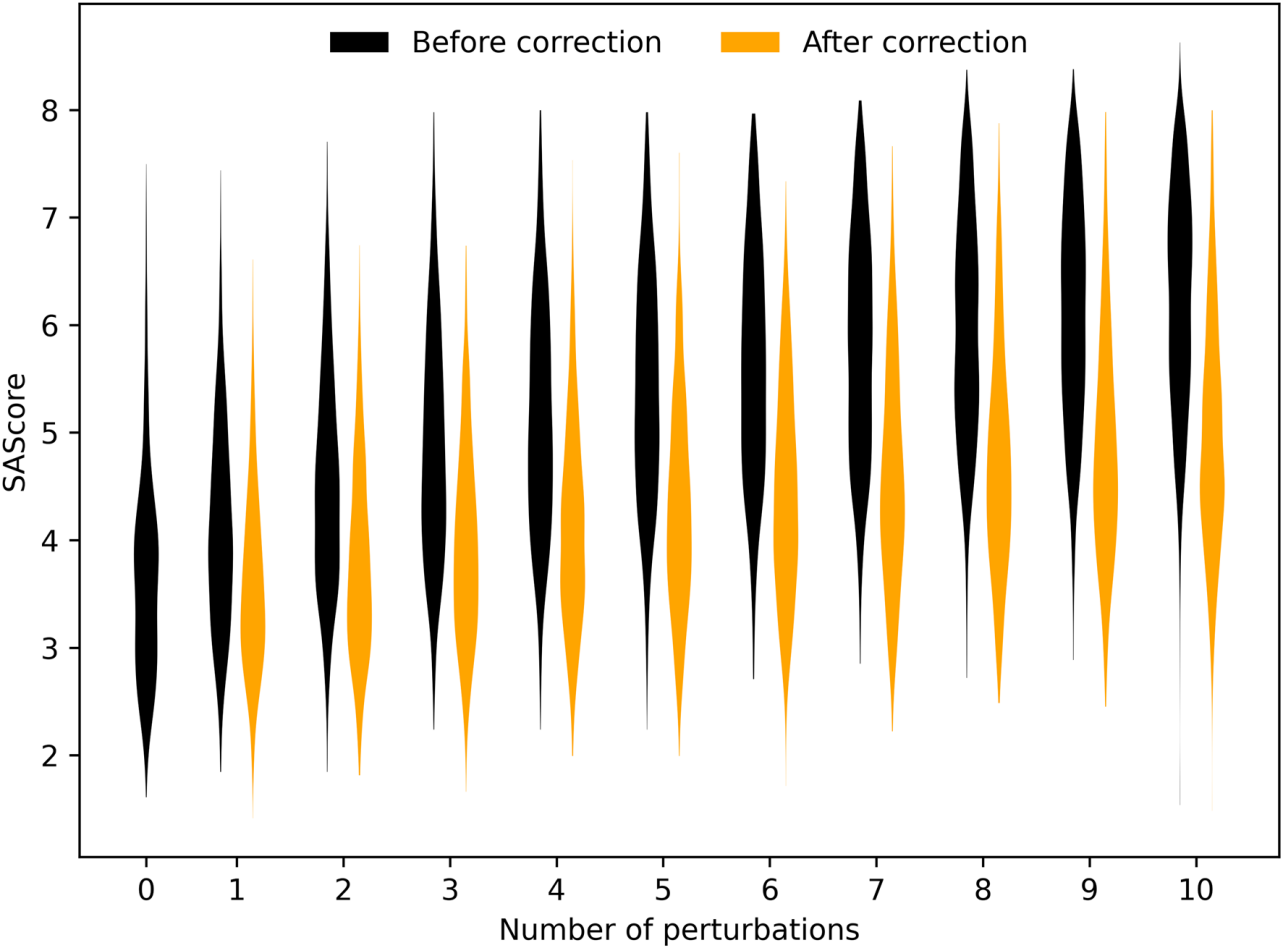
Shift in SAScore distributions associated with molecule auto-correction using the MLR selection policy. Lower SAScores are indicative of an easier synthesis. The “0 perturbations” distribution corresponds to the non-perturbed ChEMBL subset on which the perturbed molecules were based

The chemical quality of the input molecules and the output corrected molecules was assessed using the SAScore [30]. As can be seen in Fig. 11, applying random perturbations to reasonable molecules makes them progressively harder to synthesize. Encouragingly applying the correction algorithm to these broken molecules largely recovers their synthesizability. As SAScores are rather crudes measures of synthesizability [50] we sought to confirm these findings with retrosynthetic analyses [46]. Figure S5, Additional file 1 confirms that corrected molecules are indeed easier to synthesize, but for highly perturbed molecules the fraction of synthesizable molecules remains small after correction. The correction algorithm is tasked with finding a molecule that is simultaneously similar to reference chemistry and similar to the input perturbed molecule, which is by design dissimilar to reference chemistry. This is intrinsically a challenging task as both objectives are opposed. Moreover, since the retrosynthesis engine is imperfect the reported fraction of synthesizable molecules is underestimated, as exemplified by less than 60% of the ChEMBL sample being deemed synthesizable.

If the user would like to apply the algorithm in a low throughput setting, perhaps as a final sanitization step for the output of a molecule generator (Fig. 8A), we recommend choosing an explorative policy that yields molecules closely related to the input. If resources are infinite, BFS is guaranteed to yield the optimal result, but its cost scales rapidly due to the combinatorial explosion of visited chemical states as the depth of the search increases (Figure S1, Additional file 1). UCT and A* are computationally more reasonable. While both explore approximately the same number of molecules during the tree search, UCT is computationally more efficient as vertices are selected by a fast tree traversal, whereas A* requires a priority queue to be maintained. The MLR policy is a viable alternative on tight budgets. The greedy *f*_*2*_ policy can be used as fallback should all aforementioned policies fail to find solutions within reasonable amounts of time. We advise raising the ceiling on the maximum tree size as the one we chose for our benchmark is conservative. Since all molecules in the tree are stored in memory in practice the user will likely be limited by the available system memory (Figure S2, Additional file 1). Note that memory consumption will be higher when the input molecules are large.

**Fig. 12:**
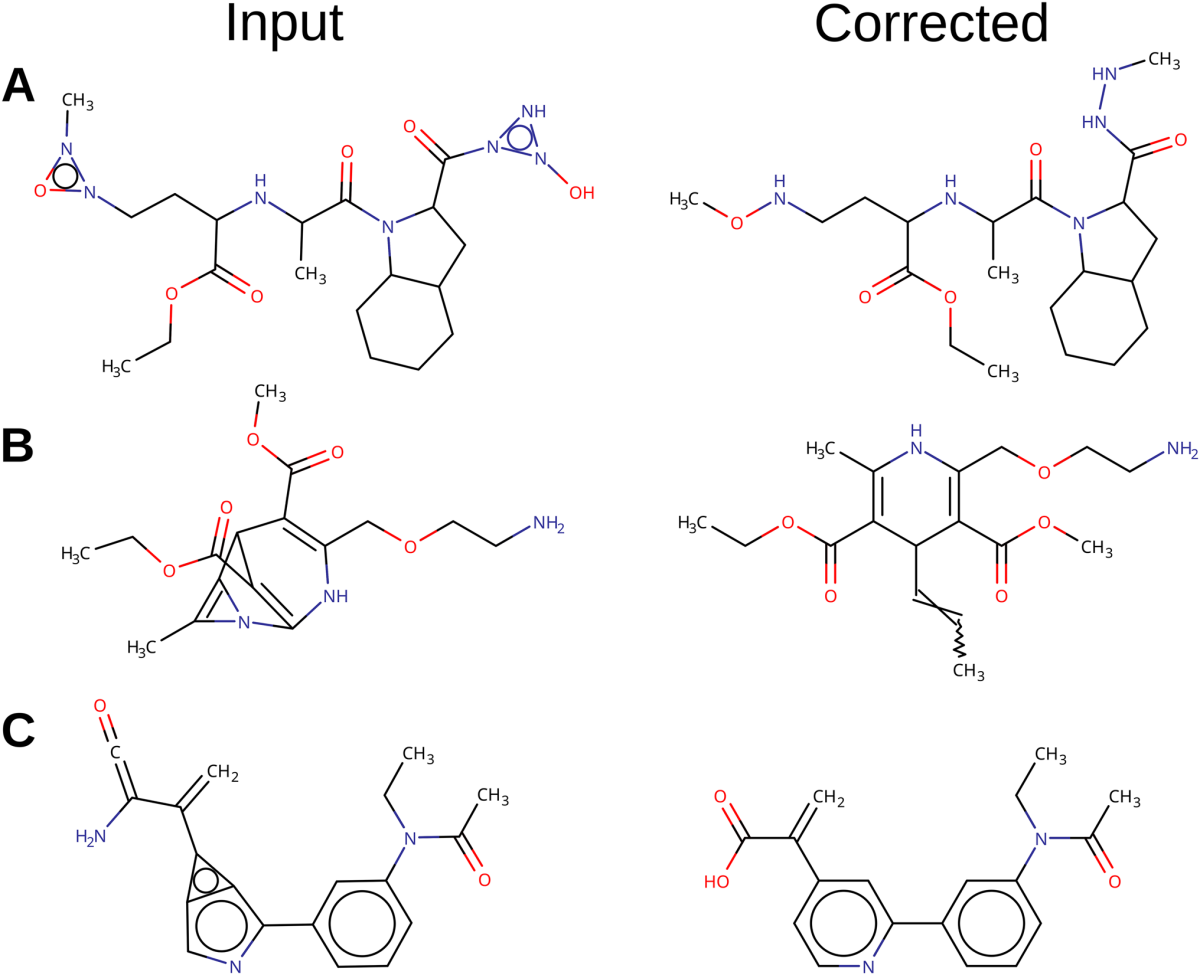
Examples of molecules designed by a naïve evolutionary algorithm (left) and their corrected counterparts (right). The MLR selection policy and a chemical dictionary with environment radii of 2 were used for correction. **A** was designed during the Perindopril MPO benchmark, **B** was designed during the Amlodipine MPO benchmark, and **C** was designed during the Sitagliptin MPO benchmark

As an example, we took molecules designed by a naive evolutionary algorithm during optimization tasks and attempted to correct them using different selection policies. A sample of incorrect molecules designed by the evolutionary algorithm as well as their corrected counterparts are shown in Fig. 12.

**Fig. 13:**
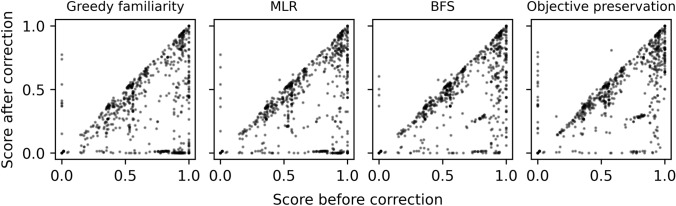
Correction algorithm’s effect on the GuacaMol benchmark scores using different selection policies. Points below the diagonal correspond to molecules becoming less fit. Molecules that were already correct are not included as their score would not change

Unfortunately, the molecules’ fitness, as assessed by the optimization task’s objective function, was degraded by the correction procedure (Fig. 13). While all policies performed reasonably well, fitness was preserved best using the explicit objective preservation selection policy. Further analysis revealed that fitness degradation was most pronounced in benchmarks whose scores depend on the presence of specific and fragile chemical features (Figure S6, Additional file 1). As one might expect the correction process can disturb these features which negatively affects the score. For a more hands-on approach to objective preservation, one could define molecular constraints to preserve key chemical features. If fitness cannot be preserved during the correction procedure through any means we recommend enforcing molecule validity throughout the construction process instead [16, 17, 36].

If the user intends to apply molecule correction iteratively to very large quantities of molecules, it is advisable to use a cheap and exploitative policy such as the greedy familiarity policy. While output molecules may not closely resemble input molecules, sometimes this is not of great importance, and sometimes it may even be beneficial. Consider a molecular design algorithm that iteratively perturbs molecules to optimize some objective function. One could attempt to correct every intermediate molecule as part of the main loop (Fig. 8B). In this case the correction would act as an integral part of the perturbation itself, essentially increasing the step size of the perturbation. This may help the algorithm in escaping local fitness minima. Even if the correction process decreases the input molecule’s fitness, the optimization algorithm would presumably correct for this by discarding the molecule, reverting to an earlier stage, or focusing its attention elsewhere. It should also be noted that if one were to correct iteratively the distances traversed by correction would match those of input molecules with a single perturbation, which are not as dramatic as those observed for highly perturbed input molecules (Fig. 9). Occasionally the correction process may effectively undo the effect of the perturbation that preceded it. While we do not anticipate this to be a large concern for most applications one could prevent it from happening using constraints.

**Fig. 14:**
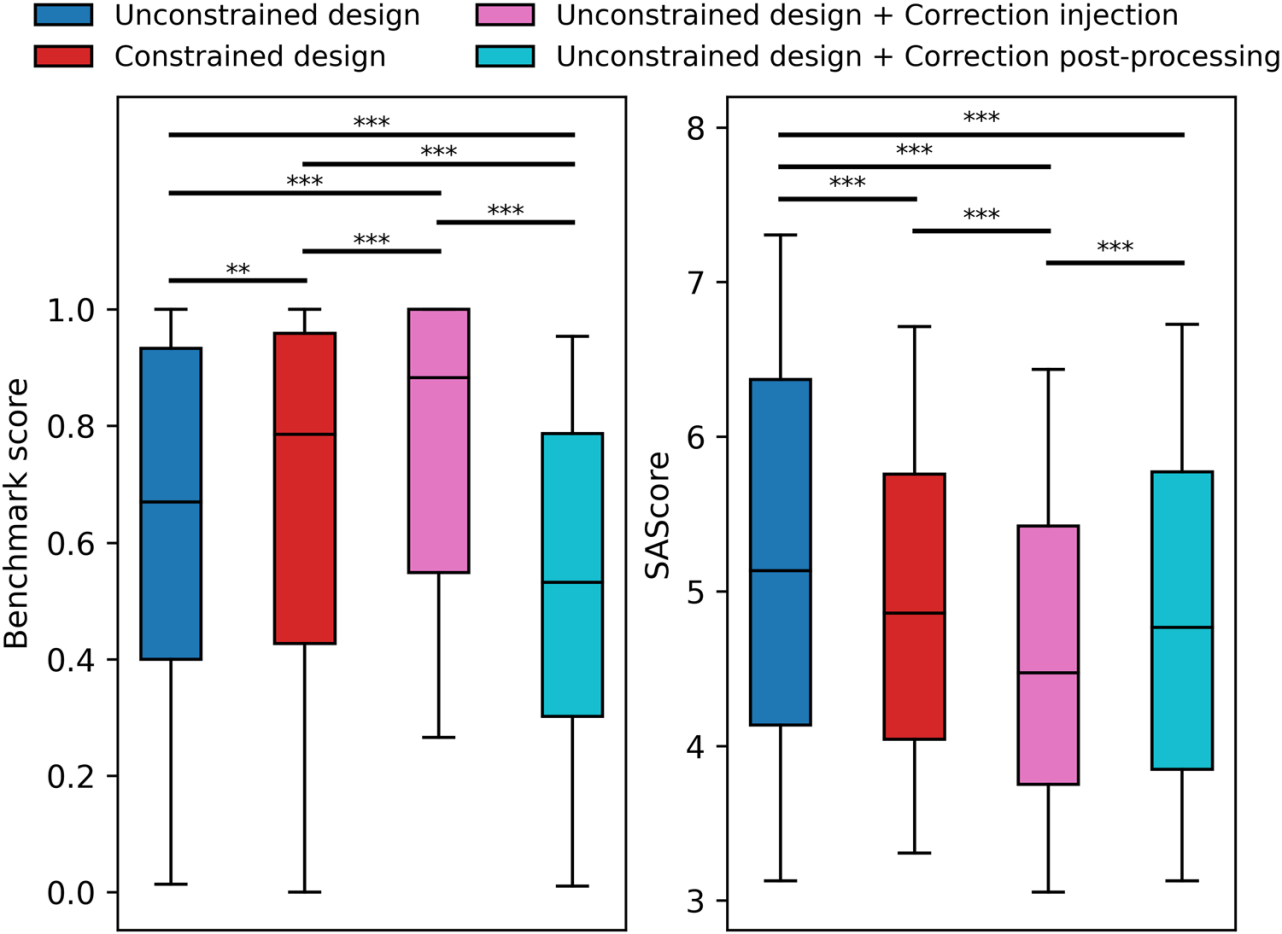
GuacaMol benchmark scores and SAScores of molecules designed by an evolutionary algorithm. Higher benchmark scores and lower SAScores are better. The objective preservation policy was used for post-processing. Unconstrained design refers to liberal modification of the molecular graph and the design of (likely) invalid molecules. All other approaches strive to design molecules with familiar circular atomic environments of topological radius 1 but achieve this goal in different ways. Constrained design refers to the use of molecular construction techniques that prevent the creation of undesirable chemical features. **: p < 0.01, ***: p < 0.001

To demonstrate the latter approach, we injected the correction algorithm into the aforementioned evolutionary algorithm (Fig. 8B). The greedy familiarity policy with a maximum tree size of merely 100 was chosen to limit computational expenses. Figure 14 shows that injecting molecule correction into existing molecule generators is a viable strategy to design molecules that are both fit and easier to synthesize compared to unconstrained molecular design. It should be noted that correction-associated synthesizability improvements are meager due to the GuacaMol benchmarking suites’ scoring functions being biased towards synthesizable molecules [3, 36]. Interestingly, iterative correction yielded better results than attempting to enforce environment correctness through molecular construction constraints (Fig. 14), and it did so consuming less computational resources (Figure S7, Additional file 1). We hypothesize that the correction procedure, being unlinked from the objective function, may drag molecules out of local fitness minima aiding the optimization algorithm in the search towards the global minimum.

For completeness’ sake the above experiments and analyses were repeated for atomic environments of radius 2. Under these conditions the correction injection approach failed to improve the synthesizability of the designed molecules, likely because the maximum tree size of 100 is insufficient to find molecules that satisfy the more stringent requirements (Figure S8, Additional file 1).

**Fig. 15:**
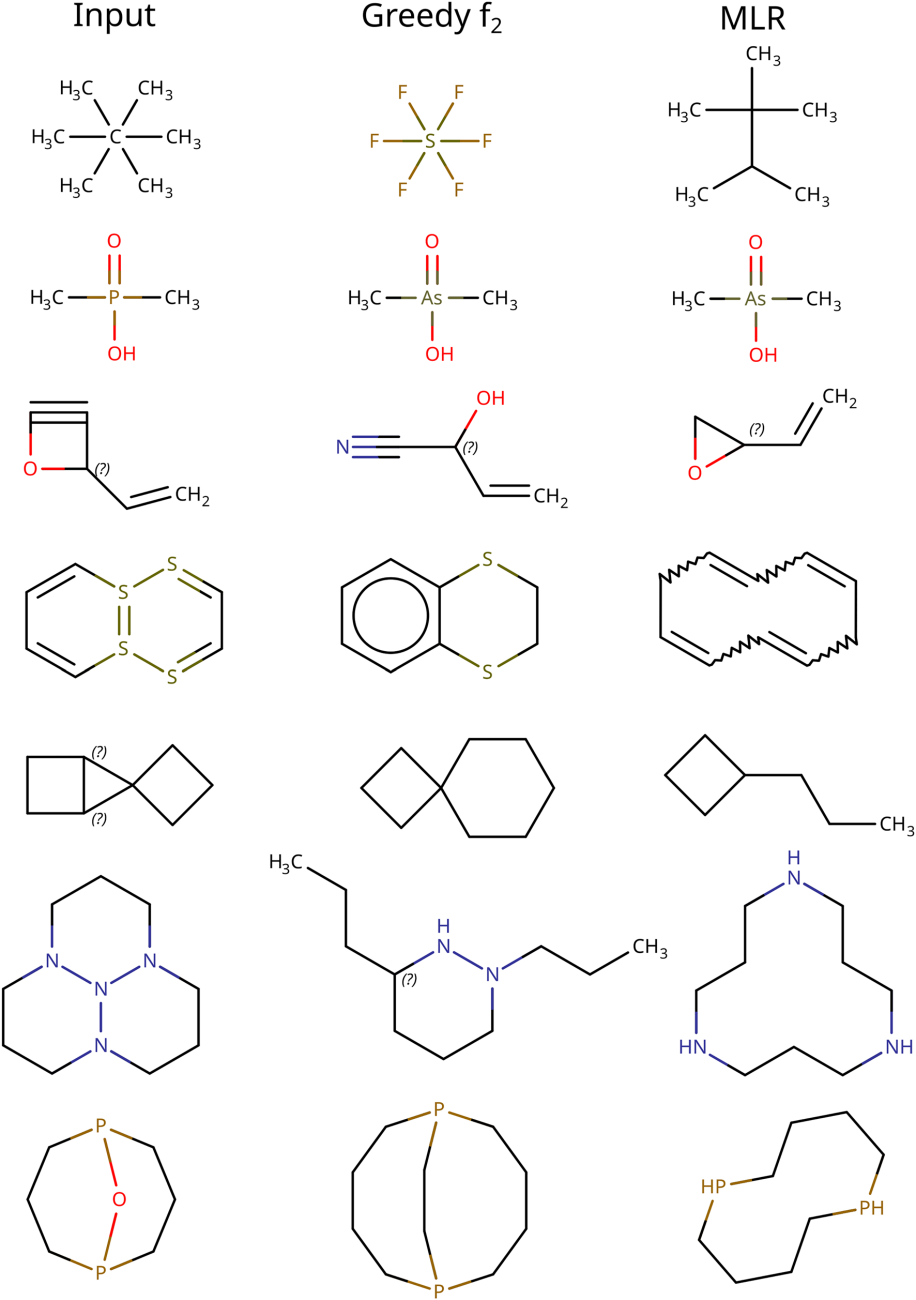
Example input molecules and their corrected counterparts using the greedy f_2_ and MLR selection policies

It should be stressed that given the same input molecule not all policies will generate the same output molecule (Fig. 15). It might be of interest to apply the algorithm with different policies and a posteriori select the most desirable output.

An unintended consequence of our expansion policy is the “carbonization” of input molecules. Perturbations most likely to increase the familiarity of a molecule are prioritized. As carbon is the backbone of organic chemistry, including our reference library of ChEMBL [10], substituting other elements with carbon is preferred by the algorithm.

**Fig. 16:**
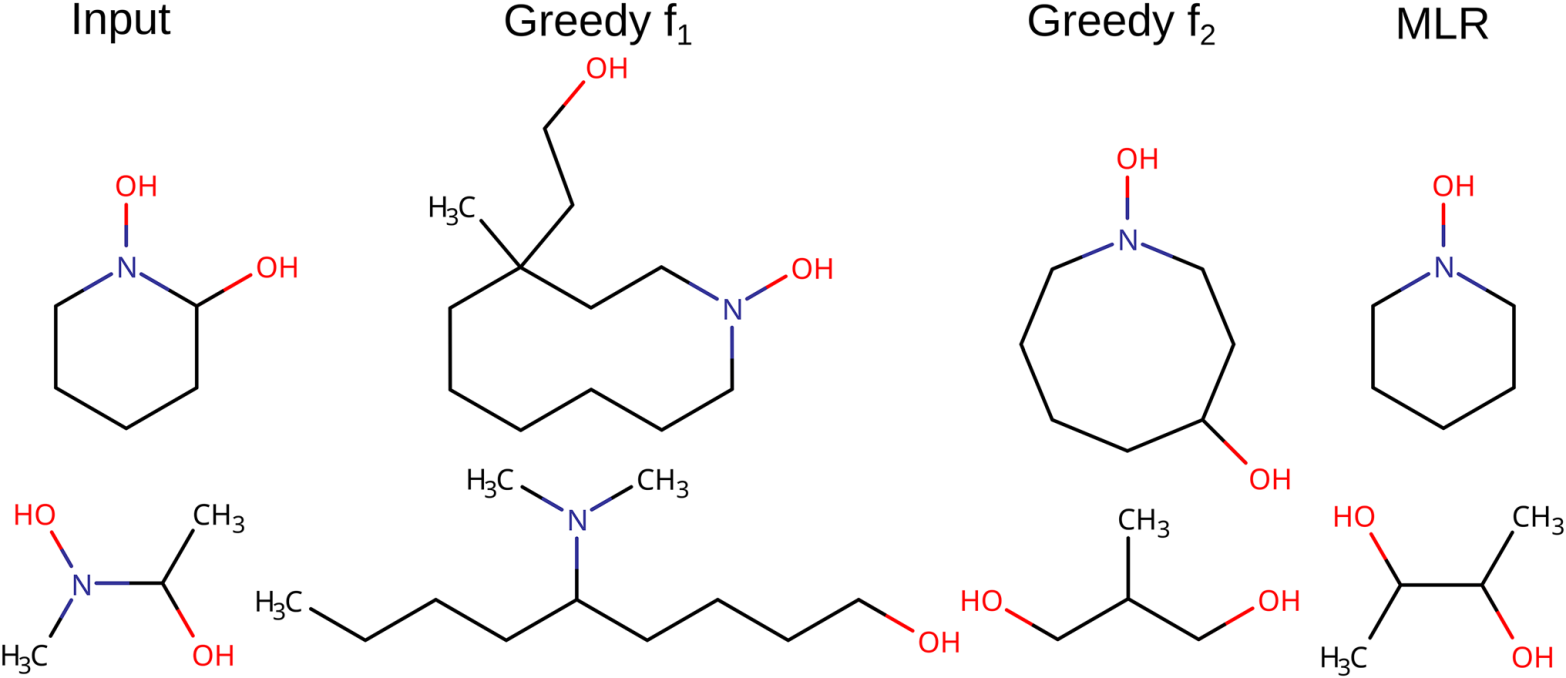
Molecule carbonization examples. The greedy f_1_ selection policy exploits the scoring function by growing long alkane chains. The other selection policies cannot exploit the scoring function in the same way, but the expansion policy still may opt to substitute heteroatoms with carbons or to separate heteroatoms by inserting carbons between them

We also encountered cases where certain selection policies would trigger the growth of long alkane chains, particularly exploitative policies such as the greedy *f*_*1*_ policy (Fig. 16, Figure S9, Additional file 1). We would like the correction process to modify existing chemical features. However, a trivial way of maximizing the *f*_*1*_ familiarity is by adding new familiar chemical features like alkanes (Eq. 3). This is a classic case of a search algorithm finding unintended ways to exploit the objective function. Frivolously adding carbons has been described previously as a strategy employed by algorithms to cheat their way to good benchmark results, be it by artificially inflating molecular diversity [4] or reaping low-hanging scoring function rewards [26, 51]. The easiest solution to the issue is to maximize the *f*_*2*_ familiarity instead (Eq. 4). While this prevents alkane growth, the search algorithm may occasionally still find it advantageous to introduce extraneous carbons as buffers between heteroatoms (Fig. 16). Correct heteroatom arrangements are tied to specific functional groups. Given a foreign functional group the path of least resistance may be to break apart said group as opposed to rearranging its atoms. The best carbonization remedy is to choose an explorative selection policy. Should this not be an option the user may choose to disable atom insertions as a perturbation or specify constraints on which parts and/or attributes of the input molecule should be preserved by the correction algorithm.

While it is possible to post-process molecules from arbitrary sources, it might not be possible to integrate the correction process into all molecule generators. We have shown how to inject it into a graph-based evolutionary algorithm, and we anticipate equivalent implementations and benefits being achievable for any molecule generators that iteratively modify molecular graphs. Integration opportunities with alternative generators are more nuanced. The algorithm’s input is a molecular graph. Our implementation is based on the RDKit [52], which means that molecules must be parsable by the RDKit to be correctable. This precludes the use of ill-formed SMILES [21]. Ill-formed SMILES can be the product of malfunctioning generative models. They may also be an intermediate state of generative recurrent neural networks [26]. In the latter case correction would have to be deferred until the SMILES string has been fully formed, potentially playing a role in sanitizing molecules prior to their objectives being evaluated. Substituting SMILES for a more robust line notation such as SELFIES [23] whose intermediate strings are also valid would enable the “auto-correct” process to behave more as a molecule “auto-complete”. In any case the correction process would play a role in steering the chemical space search. Whether this would antagonize or synergize with the model’s inherent guidance remains to be explored.

Caution should be applied when employing molecule generators that rely on the similarity principle, for they amplify existing chemical biases in data due to prior art data conditioning future data collection [17, 53]. This can have detrimental effects on chemical novelty. The problem is compounded by building pipelines of tools relying on the same principle, as we do in this work. We are aware this is suboptimal, but in absence of competing methods grounded on physical first-principles, chemical bias amplification postures itself as a necessary evil.

One area worth revisiting in the future is the way in which correctness is assessed. Currently molecular keys are considered either foreign or familiar, depending on their frequency in the chemical dictionary. While the frequency threshold separating both categories can be tweaked, it would be preferable to treat familiarity as a frequency-dependent continuous variable. We also believe there is potential in further development of selection policies. The policies explored herein rely on crude heuristics. We can draw inspiration for policy design from other fields where tree searches are used. Synthesis planning in particular has recently witnessed major breakthroughs thanks to machine learning augmented policies [54, 55]. We believe that similar methods could be applied here to better direct the search, reducing the risk of missing good solutions as well as the cost to find said solutions.

## Conclusions

We present an algorithm that can identify and fix problems within molecular graphs. It is implemented as a tree search that iteratively modifies input molecules until it yields a correct molecule. Strategies to minimize the length of the search and maximize the similarity between input incorrect and output corrected molecules were developed. To the best of our knowledge this is the first algorithm of its kind, opening the door to novel workflows in molecular design. The algorithm can be used to post-process the output of molecule generators, possibly salvaging molecules that would have otherwise been discarded due to chemical quality concerns. It may also be integrated into faulty molecule generators to patch some of their shortcomings or even augment their capabilities. Ultimately, it enables researchers to delegate chemical quality assurance to the algorithm instead of engineering new systems from scratch. We hope the algorithm will economize computational and human resources in molecular design.

## Supplementary Information

Below is the link to the electronic supplementary material.

Supplementary file1 (PDF 883 kb)

## Data Availability

Project name: MoleculeAutoCorrect. Project home page: https://github.com/AlanKerstjens/MoleculeAutoCorrect. Archived version: MoleculeAutoCorrect_0.0.2. Operating system(s): Platform independent. Programming language(s): C +  + , Python. Other requirements: RDKit cheminformatics toolkit [52], Molpert [36]. License: AGPL 3.0.
