# Supplementary material for: Molecule auto-correction to facilitate molecular design
Source: J Comput Aided Mol Des. 2024 Feb 16;38(1):10. doi: 10.1007/s10822-024-00549-1 (PMC10873457; doi:10.1007/s10822-024-00549-1)
Supplement: Supplementary file 1 — Supplementary file1 (PDF 883 kb) [file 10822_2024_549_MOESM1_ESM.pdf]

Molecule auto-correction to facilitate molecular design: Additional file 1

Alan Kerstjens and Hans De Winter

Laboratory of Medicinal Chemistry, Department of Pharmaceutical Sciences, University of Antwerp

Universiteitslaan 1, 2610 Wilrijk, Belgium

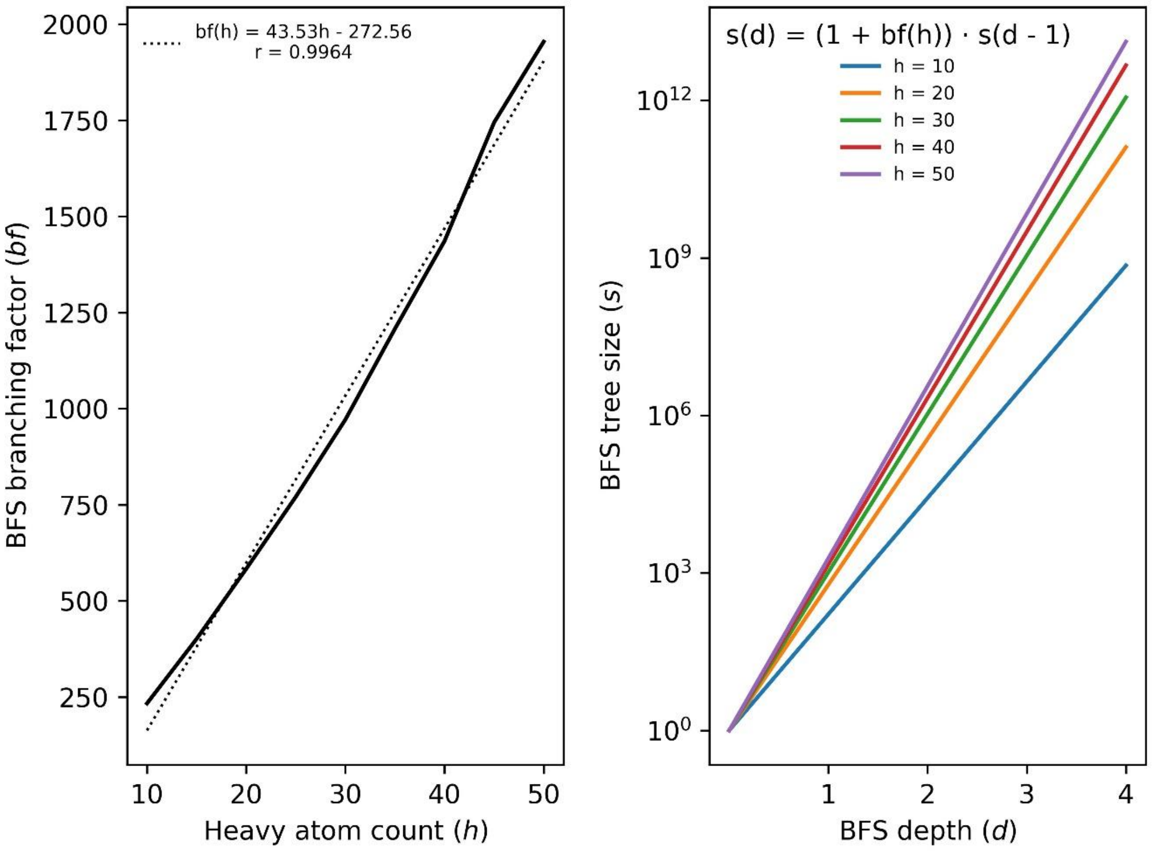

Figure S1. Left panel: Branching factor (*bf*) of a Breadth-First Search (BFS) as a function of the root molecule's number of heavy atoms (*h*). The branching factor was calculated by enumerating all neighboring molecules using Molpert's "balanced" settings. Right panel: Projection of tree size (*s*) for a given BFS depth (*d*) assuming constant molecule size throughout the search. This assumption is reasonable since the average heavy atom count of molecules only increases about 0.25 per BFS search level.

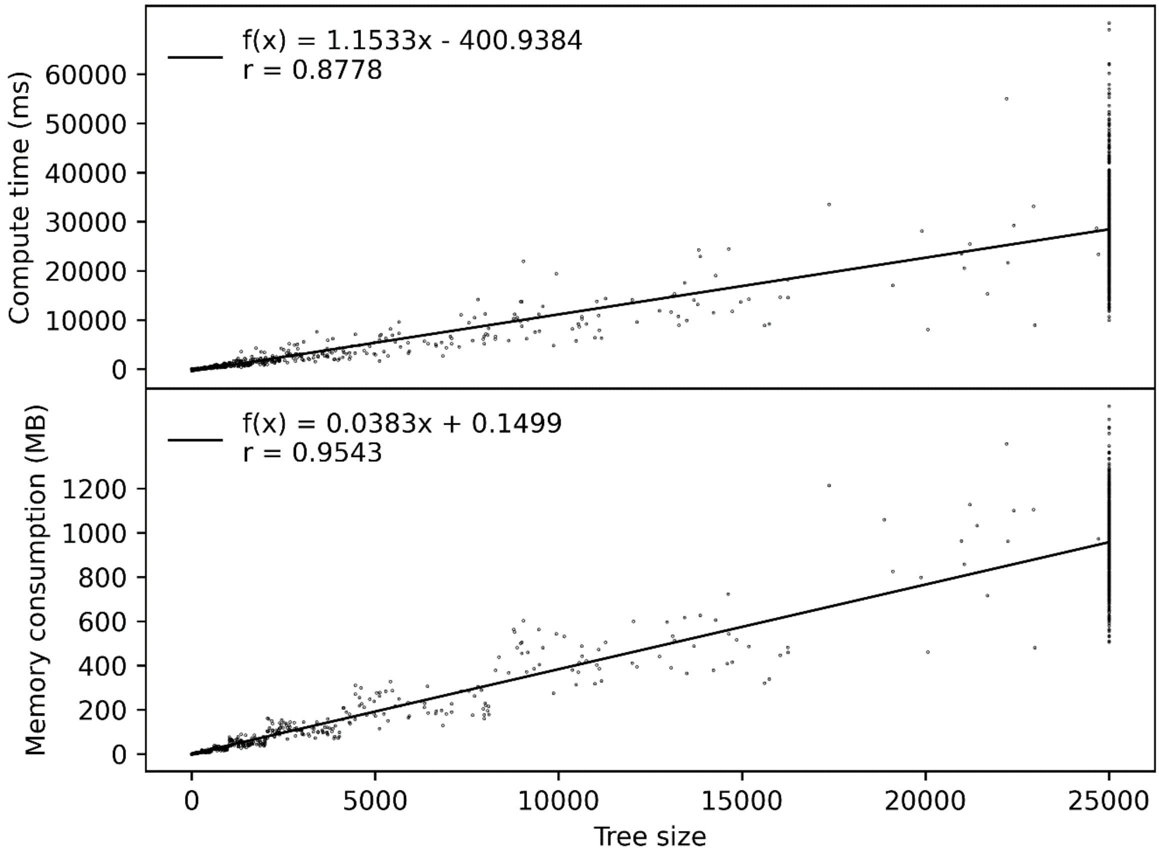

Figure S2. Correlation between tree size, search compute time and memory consumption. Compute time is given for a single-threaded workload on an AMD Epyc 7452 CPU @ 2.35 GHz.

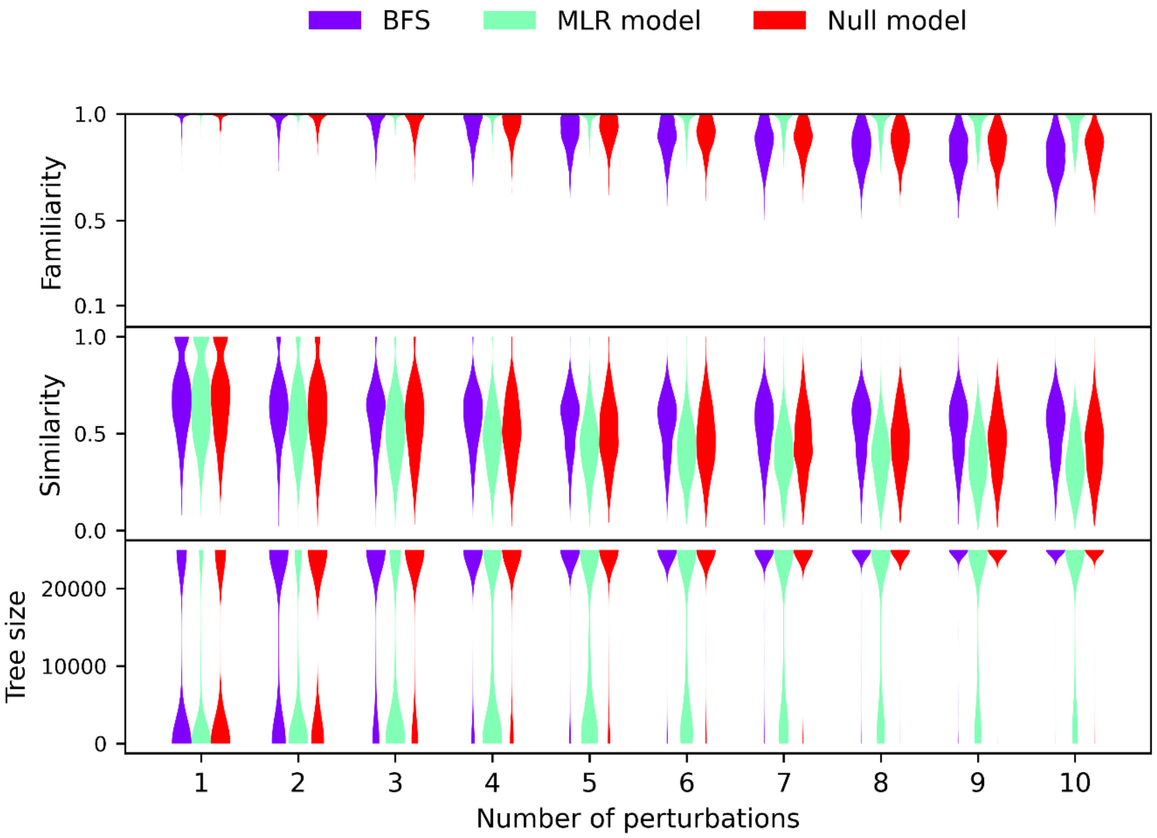

Figure S3. Comparison between the MLR model and its null equivalent during the molecule correction benchmark. The number of perturbations applied to the input molecule is shown on the x axis. The violin plots display the density of output molecules' properties and the cost to generate them. Note that the tree size was limited to a maximum of 25000.

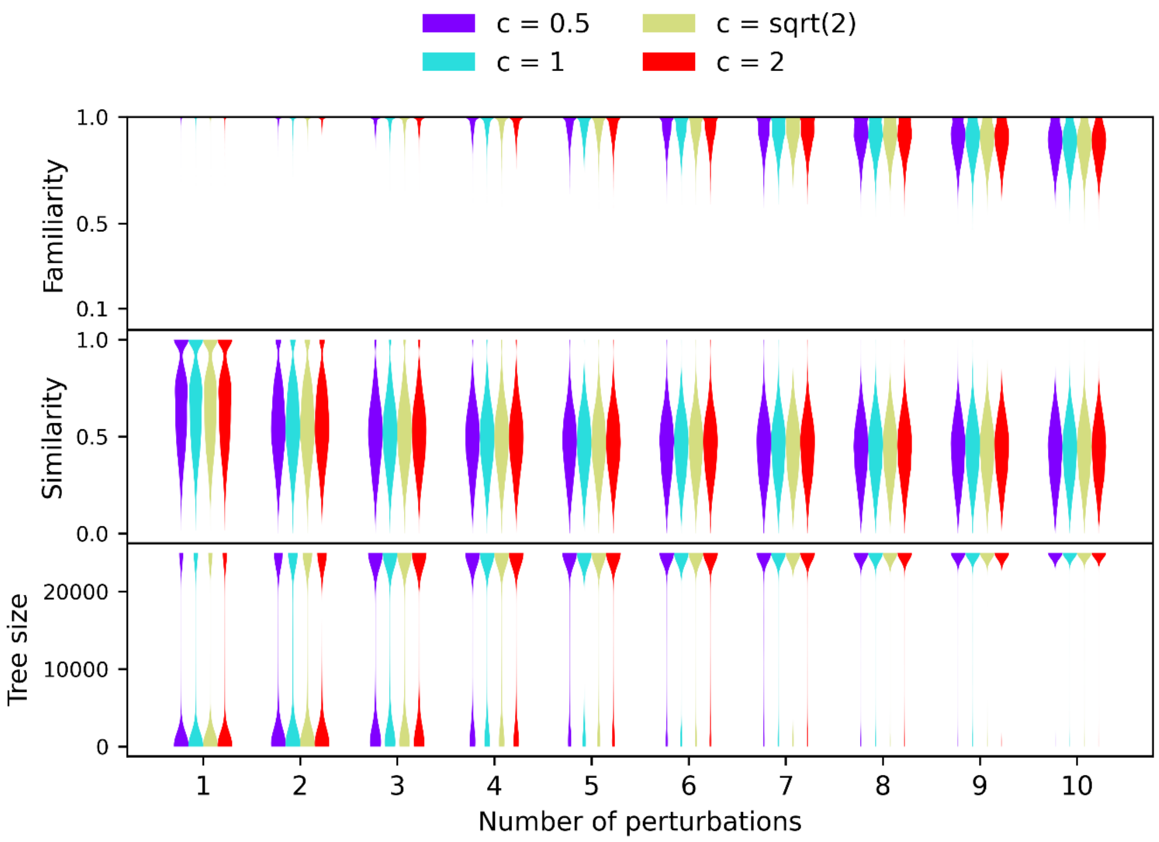

Figure S4. Molecule correction benchmark results using the UCT selection policy with different *c* coefficients. The number of perturbations applied to the input molecule is shown on the x axis. The violin plots display the density of output molecules’ properties and the cost to generate them. Note that the tree size was limited to a maximum of 25000.

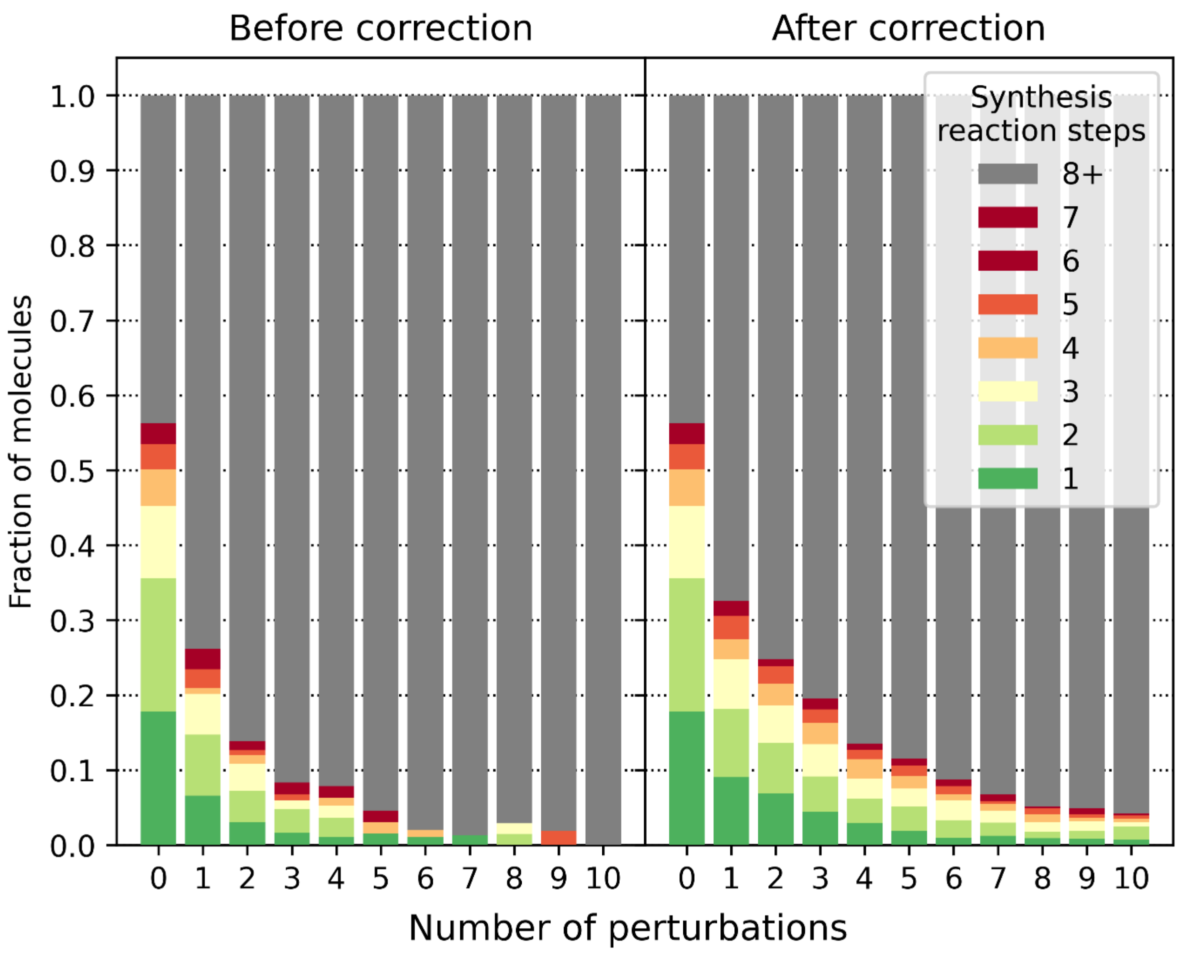

Figure S5. Fraction of molecules synthesizable within a certain number of synthetic steps based on the number of random perturbations they were subjected to and whether they were corrected or not. The MLR selection policy and a dictionary with atomic environment radii of 2 were used for the correction process. Retrosynthetic analyses were performed using AiZynthFinder using the ZINC reactant stock and USPTO-derived reaction template policy provided by the authors. 0 perturbations corresponds to the control ChEMBL sample. Molecules requiring 8 or more synthetic steps are considered unsynthesizable.

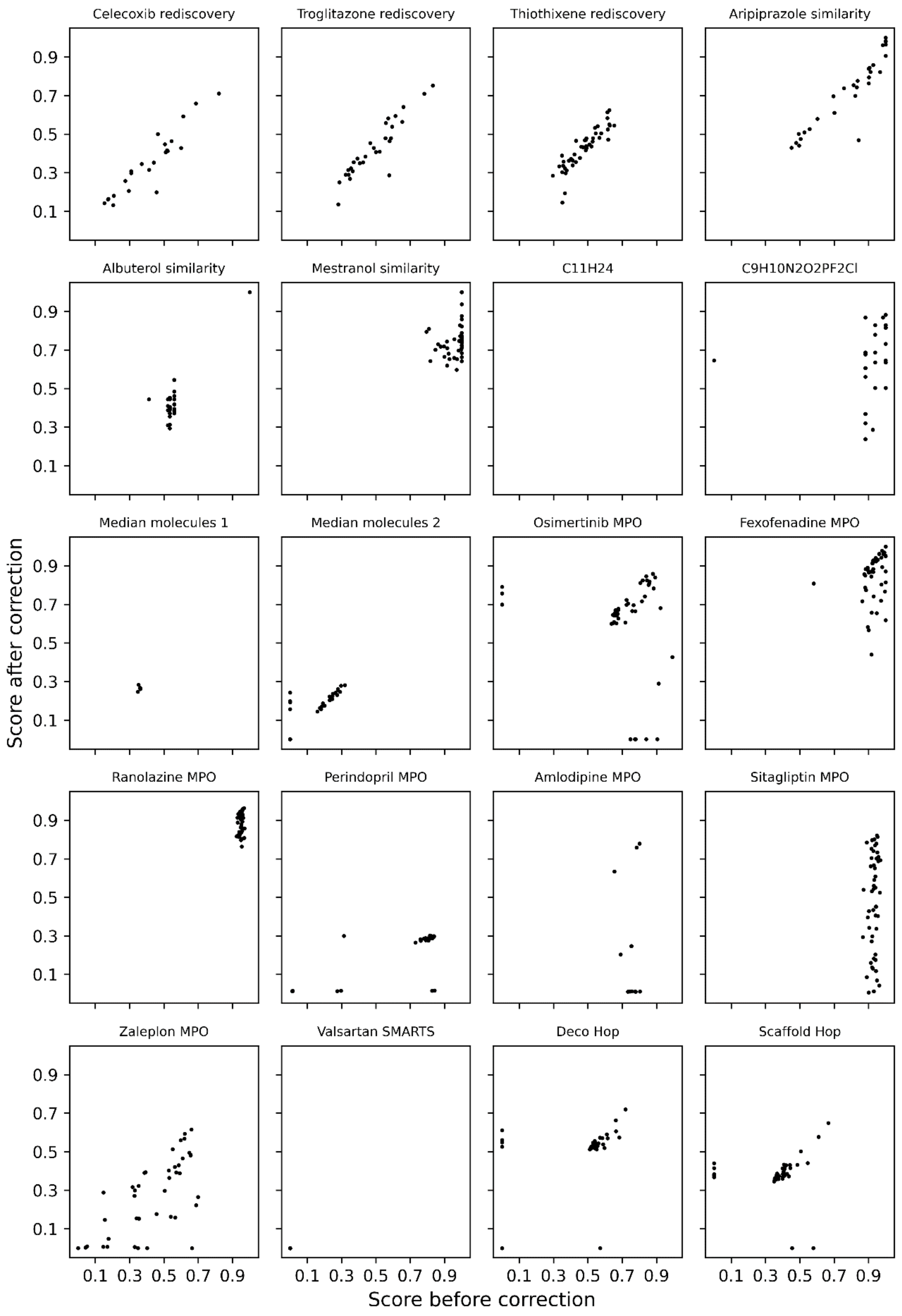

Figure S6. GuacaMol benchmark suite score degradation broken down per benchmark. Explicit objective preservation was used as selection policy. Molecules that were already correct aren't included. Benchmarks showing the sharpest score degradation are dependent on specific chemical features and sensitive to molecular modifications. For example, C9H10N2O2PF2Cl, Ranolazine MPO and Sitagliptin MPO require the presence of infrequent elements such as halogens or phosphorus, which may be removed or substituted by the algorithm. Perindopril MPO and Amlodipine MPO require the presence of specific numbers of (aromatic) rings, which are easily broken.

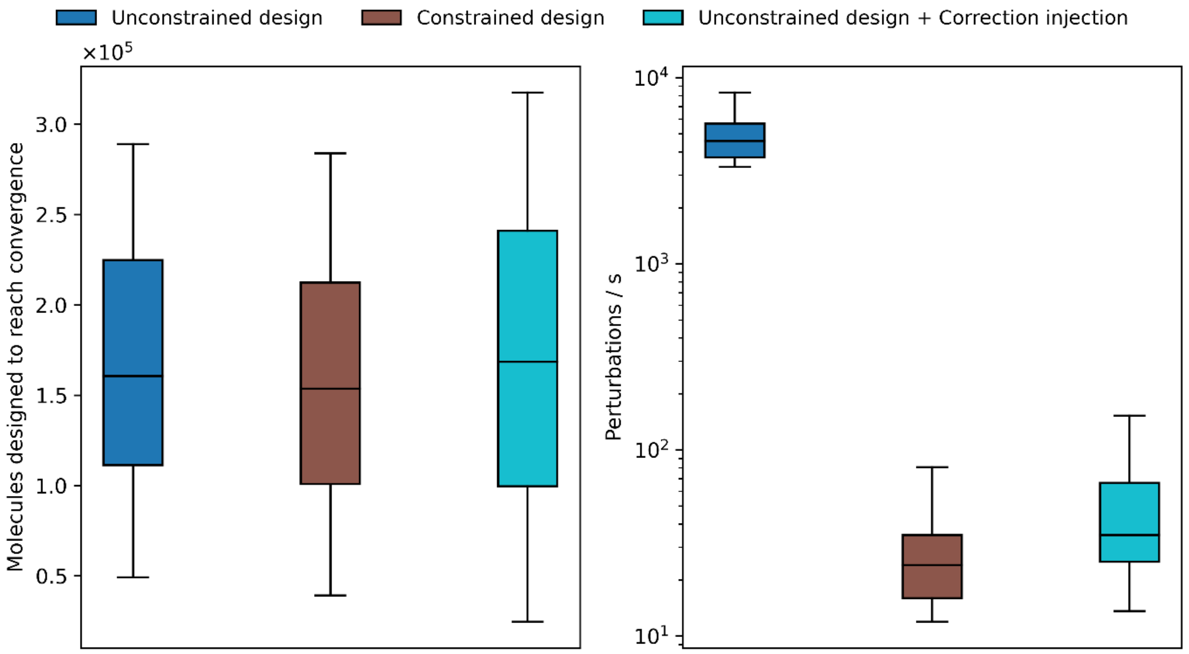

Figure S7. Computational cost of designing molecules using different variants of the same evolutionary algorithm. Unconstrained design is the fastest but may result in chemically invalid molecules. The two other approaches both result in molecules with familiar atomic environments of radius 1. Despite achieving this goal in different ways their cost is comparable. Timings are given for a single-threaded workload on an AMD Epyc 7452 CPU @ 2.35 GHz.

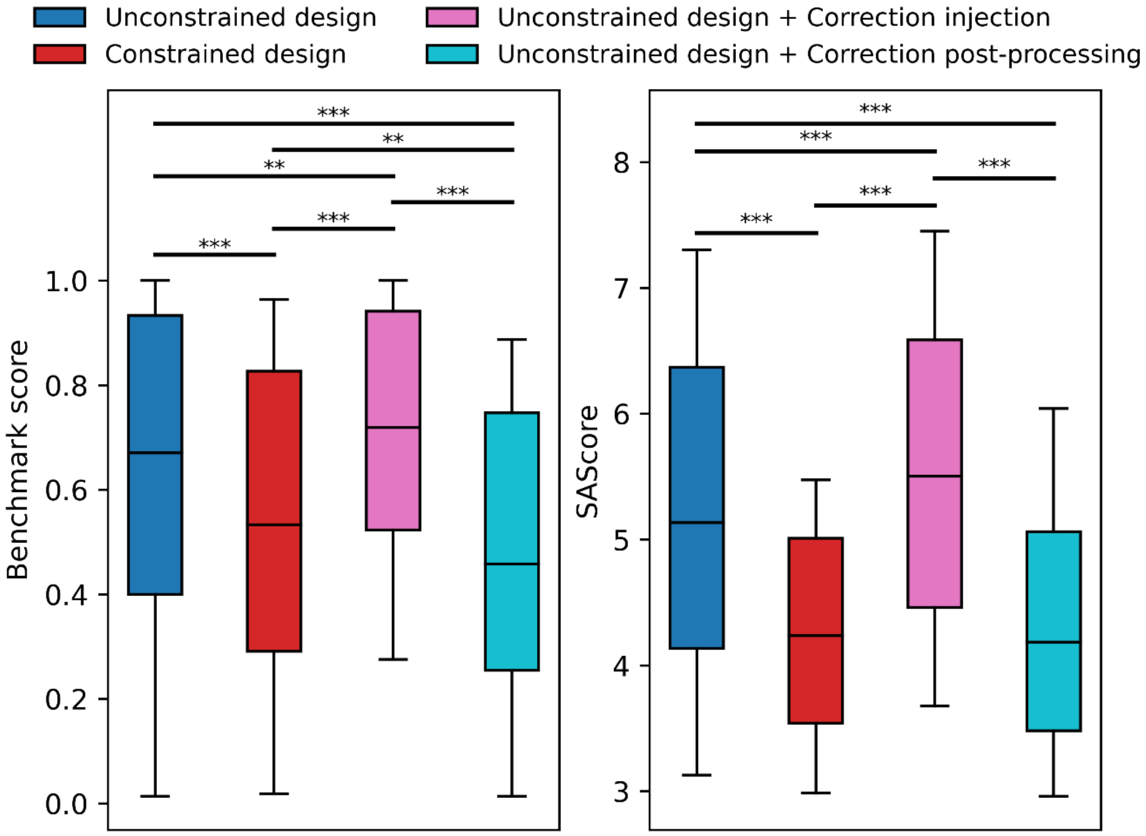

Figure S8. Benchmark scores and SAScores of molecules designed by an evolutionary algorithm. This figure is analogous to Figure 14. In both cases the designed molecules were forced to exhibit familiar circular atomic environments, with the key difference being the radii of said environments: 1 for Figure S14 and 2 for the present figure. The objective preservation policy was used for post-processing. Unconstrained design refers to liberal modification of the molecular graph and the design of (likely) invalid molecules. Constrained design refers to the use molecular construction techniques that prevent the creation of undesirable chemical features. **: $p < 0.01$, ***: $p < 0.001$.

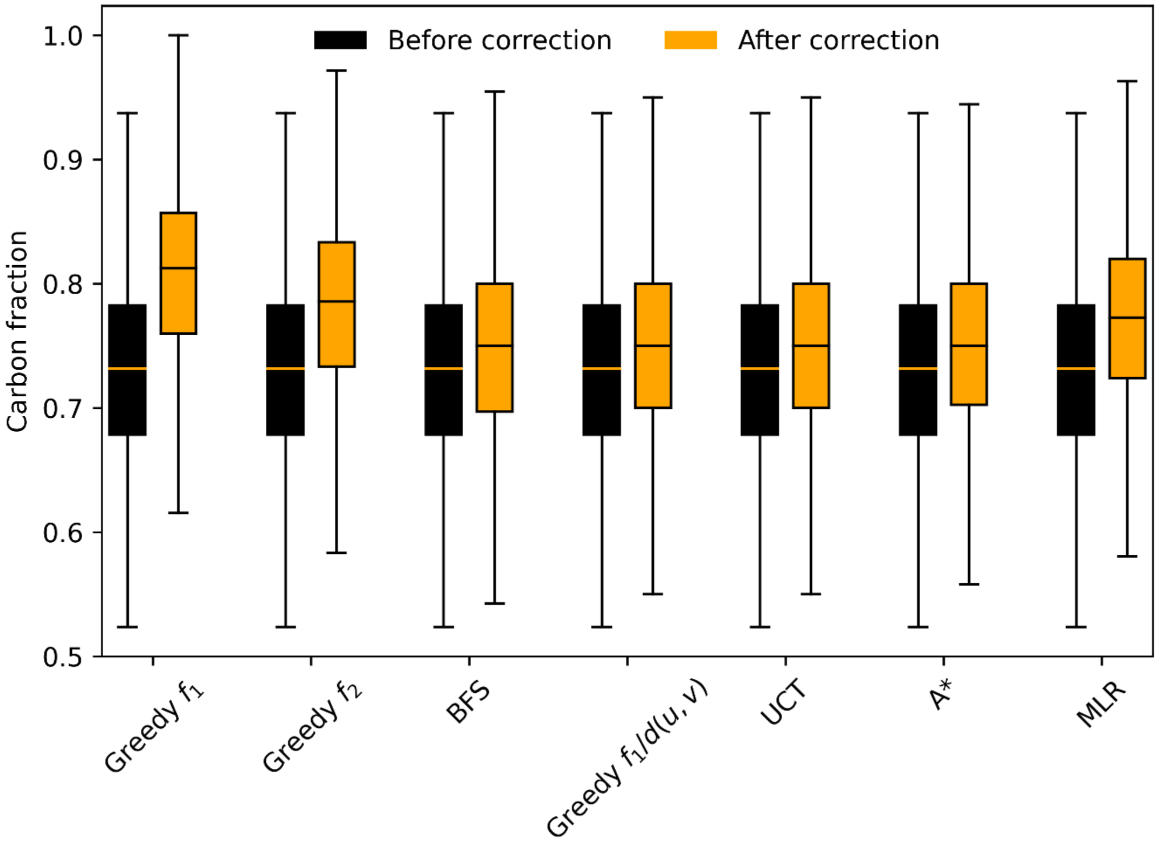

Figure S9. Fraction of a molecule's atoms that are carbons, before and after molecule correction using different selection policies. The most exploitative selection policies increase the carbon fraction the most.
